# Prevalence and Concentration of Mycotoxins in Animal Feed in the Middle East and North Africa (MENA): A Systematic Review and Meta-Analysis

**DOI:** 10.3390/toxins15030214

**Published:** 2023-03-10

**Authors:** Ghader Jalilzadeh-Amin, Bahram Dalir-Naghadeh, Masoud Ahmadnejad-Asl-Gavgani, Aziz A. Fallah, Amin Mousavi Khaneghah

**Affiliations:** 1 Department of Clinical Pathology and Internal Medicine, Faculty of Veterinary Medicine, Urmia University, Urmia 5756151818, Iran; 2 Department of Food Hygiene and Quality Control, Faculty of Veterinary Medicine, Shahrekord University, Shahrekord 8818634141, Iran; 3 Department of Fruit and Vegetable Product Technology, Prof. Wacław Dąbrowski Institute of Agricultural and Food Biotechnology–State Research Institute, 36 Rakowiecka St., 02-532 Warsaw, Poland; 4 Department of Technology of Chemistry, Azerbaijan State Oil and Industry University, 16/21 Azadliq Ave, Baku AZ1010, Azerbaijan

**Keywords:** mycotoxins, meta-analysis, feed, MENA

## Abstract

This study seeks a comprehensive meta-analysis of mycotoxin contaminants in animal feed consumed in the Middle East and North Africa (MENA) region. The obtained articles were reviewed, and 49 articles that investigated the contamination of mycotoxins including aflatoxins (AFs), deoxynivalenol (DON), zearalenone (ZEA), T-2 toxin, fumonisins (FUM), and ochratoxin A (OTA), in feed samples or components of animal feed in the MENA region were selected. The titles of the final articles included in the study were meta-analyzed. Necessary information was extracted and categorized from the articles, and a meta-analysis was performed using Stata software. The highest contamination was in dry bread (80%), and Algeria was the most contaminated country (87% of animal feed), with the most mycotoxins contaminating AFs (47%) and FUM (47%). The highest concentration of mycotoxins in animal feed is related to FUM (1240.01 μg/kg). Climate change, economic situation, agricultural and processing methods, the nature of the animal feed, and improper use of food waste in animal feed are among the most critical factors that are effective in the occurrence of mycotoxin contamination in animal feed in MENA. Control of influential factors in the occurrence of contaminations and rapid screening with accurate identification methods to prevent the occurrence and spread of mycotoxin contamination of animal feed seem important.

## 1. Introduction

The mycotoxins are secondary toxic metabolites of certain species of fungi, including *Aspergillus*, *Fusarium*, and *Penicillium*. These toxins are produced by pathogenic molds and infect plants or crops [1,2,3,4,5,6,7]. Various mycotoxins have been identified from animal feed, and studies have shown that most feed samples are contaminated with at least one mycotoxin [8]. However, aflatoxins (AFs), deoxynivalenol (DON), zearalenone (ZEA), T-2 toxin, fumonisins (FUM), and ochratoxin A (OTA) are the main mycotoxins contaminating animal feed and are considered dangerous to animals’ health [9,10,11,12]. Moreover, the presence of mycotoxins in animal products could be a public health hazard [13,14]. Therefore, animal feed safety is an essential prerequisite for human food safety, and the slogan “Feed for Food” clearly shows the importance of this issue [15,16].

Exposure to AFs in animal feed can reduce milk production, reproductive performance, and immune function and can increase susceptibility to various diseases in livestock [17]. Intake of FUM can affect animals’ nervous, renal, hepatic, reproductive, and digestive systems, causing oxidative stress and inducing apoptosis [18]. In addition to being toxic to the immune system and genes, ZEN can have estrogenic effects and cause endocrine, reproductive, and growth disorders [19]. Trichothecenes (TCTs), such as T-2 and HT-2 toxins, and DON (or vomitoxin) can be absorbed from the gastrointestinal tract or even topically and inhalational and adversely affect the liver, kidneys, skin, gastrointestinal, reproductive, neuroendocrine, immune, lymphoid, and hematopoietic systems. Their pathogenesis pathway is inhibition of protein synthesis, changes in intestinal microbiota, induction of oxidative stress, inflammation, and apoptosis. Decreased appetite, growth retardation, and gastroenteritis are symptoms of TCT poisoning in animals [20,21,22].

Cows receiving diets contaminated with AFB1, ZEA, and DON had significantly altered γ-glutamyl transpeptidase, total antioxidant capacities, and serum metabolites involved in amino acid metabolism [23]. DON- and FUM-contaminated TMR feed in dairy cows reduce milk quality, dietary digestibility, immune system function, and metabolic profile disorders [24]. The presence of mycotoxins in pig feed can reduce feed intake by 18% and weight gain by 21%, and DON had the most significant effect on this reduction. Young animals, males, and those receiving the highest concentrations of mycotoxins were the most affected. Mycotoxins also affect the relative weight of pigs’ internal organs, such as kidneys, liver, and heart [25]. Aflatoxins can cause reduced productivity, immune system dysfunction, hepatic injury, and even mortality in broilers. Additionally, feeding with Afs-contaminated feed can have an adverse effect on the feed conversion ratio of broilers at the end of the first week (low), second week (moderate), and third to sixth week (very high) [23]. The presence of mycotoxins in the diet of broilers causes significant hematologic changes in hematocrit, hemoglobin, leukocytes, heterophils, and lymphocytes and significant biochemical changes in creatine kinase, alkaline phosphatase, alanine aminotransferase, aspartate aminotransferase, creatinine, triglycerides, albumin, globulin, cholesterol, total protein, calcium, and mineral phosphorus [26]. Intestinal cells are among the first to be exposed to ingested mycotoxins, so they are exposed to high concentrations of mycotoxins. Mycotoxins can reduce the surface available for nutrient absorption, disrupt nutrient transport and intestinal barrier function, and perpetuate intestinal pathogens and intestinal inflammation [27].

The animal feed includes livestock, horse, fish, and poultry feed, formulated to ensure the supply of nutrients needed to maintain the health and proper performance of animals. Plant and animal materials are used to meet these needs in animal feed. Both sources of animal feed can pose biological, chemical, and physical hazards to the animals [28]. Animal feed directly affects livestock health and welfare. It is an important part of the food chain that directly plays a role in the safety of animal-origin foods and, consequently, human health [29]. Harmful effects of mycotoxins include liver toxicity, teratogenicity, mutagenicity, neurotoxicity, skin toxicity, carcinogenicity, estrogenicity, and immunosuppressive effects [30,31,32]. For example, aflatoxin B1 (AFB1)-contaminated animal feed is converted to aflatoxin M1 (AFM1) by the cytochrome p450 associated enzyme 15 min after ingestion, and approximately 0.3–6.2% of the consumed AFB1 enters the milk as AFM1 [32,33,34,35]. The transmission of feed AFB1 to milk AFM1 depends on the breeding system, milk production status, animal health, and diet [36,37].

Materials used to feed livestock, processed, semi-processed, or unprocessed, are called animal feed. The composition of animal feed is different in countries and even livestock farms. However, in general, cereals and cereal-based products are animal feed’s most commonly used ingredients. Most of the world’s corn production (55%) and about 20% of the total wheat area are allocated to animal feed [14]. In cereal processing, mycotoxins are mainly used for animal feed [38].

Allergies to mycotoxins are a significant concern for those who are exposed to them, either through the consumption of contaminated food or exposure to mold in indoor environments [39,40]. However, allergies to mycotoxins in animal feed are relatively rare but can occur in animals with a hypersensitive immune system. These allergies can manifest in various ways, such as respiratory problems, skin irritation, and digestive issues, which can lead to reduced growth rates, weight loss, and in severe cases, mortality [41,42,43,44,45,46]. To prevent allergies to mycotoxins in animal feed, it is essential to maintain proper storage conditions, regularly test feed for mycotoxin contamination, and use appropriate detoxification techniques before feeding animals. Additionally, early detection and prompt treatment of any allergy symptoms can help minimize the impact on animal health and productivity [47,48,49,50].

Elimination of contaminated feed reduced livestock productivity, and costs of veterinary care are parts of the economic impact of mycotoxin contamination of animal feed on the livestock industry [51]. One short-term direct financial loss related to AFs contamination in maize in the Netherlands in 2013 was estimated at between € 12 and € 25 million, of which 60% was for traders, 39% for the feed industry, and 1% for the dairy industry [52]. Contamination of animal feed and its components also disrupts international trade [53].

The occurrence of feed mycotoxins varies in different geographical areas [38]. The results of a study have shown that animal feed in the Middle East and North Africa (MENA) region has also been significantly contaminated with mycotoxins such as AFs, TCT, ZEN, FUM, and OTA [42,54]. Moreover, the occurrence of AFM1 in raw, pasteurized, and ultra-high temperature processing (UHT) milk is also high in MENA, which can even increase the risk of cancer in children. The high level of AFM1 may reflect the high contamination of animal feed with mycotoxins in the MENA region [55].

Meta-analysis is a method applied to integrate data from published individual studies and can provide new, comprehensive, and valuable results [56]. Meta-analysis is used in the fields of human medicine and veterinary medicine [57,58,59,60,61,62], food [56,63,64,65,66], and feed safety [67,68,69].

Due to the unavailability of a comprehensive study of the occurrence and levels of mycotoxins in animal feed in the MENA region, the present study was performed on the subject through a systematic review and meta-analysis.

## 2. Results

The number of articles retrieved from significant database searches for search combinations between 2010 and 2022 was 2632. The number of articles obtained from each database was as follows: PubMed 316, Web of Science 918, Scopus 870, Embase 368, and Google Scholar 160. After importing all the results into the EndNote software, 955 articles were identified as duplicates and deleted. Of the remaining articles, 377 were identified and deleted as duplicate articles in the manual screen of reviewers. After evaluation of the title and abstract of the articles, 1064 articles were found to be inappropriate and were removed. In the search for the full text of the remaining articles, no complete text was found for 108, and they were left out of the study. Finally, out of 132 full-text articles reviewed, 49 were identified as eligible and entered into the meta-analysis (Table S1) [70,71,72,73,74,75,76,77,78,79,80,81,82,83,84,85,86,87,88,89,90,91,92,93,94,95,96,97,98,99,100,101,102,103,104,105,106,107,108,109,110,111,112,113,114,115,116,117].

Most studies were conducted in Asian countries (265/323, 82%) and fewer in African countries (58/323, 17.9%). The majority of trials were in Pakistan (116/323, 35.9%) and Iran (52/323, 16.1%); the remaining were in Turkey (48/323, 14.9%), Egypt (31/323, 9.6%), Tunisia (21/323, 6.5%), Qatar (18/323, 5.6%), MENA countries (16/323,5%), Saudi Arabia (11/323, 3.4%), Jordan (3/323, 0.9%), Sudan (3/323, 0.9%), Algeria (2/323, 0.6%), Morocco (1/323, 0.3%), and Yemen (1/323, 0.3%) (Table 1). The proportional independent study was not conducted in Iraq, Afghanistan, Armenia, Azerbaijan, Bahrain, Cyprus, Djibouti, Georgia, Israel, Kuwait, Lebanon, Libya, Malta, Mauritania, Oman, Palestine, Somalia, and Syria.

**Figure 1:**
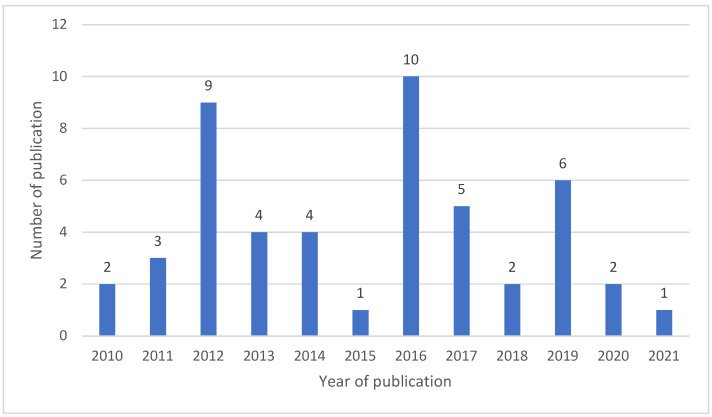
Number of studies on aflatoxins in animal feed in the MENA region from 2010 to 2021.

Most studies were published in 2016 with 10 articles. A total of 33 out of 49 studies were published between 2010 and 2016, and there have been only 16 studies in the last 5 years (Figure 1). The sample size in the published studies was 18,748. Among the 323 studies, 172 were related to AFs (53.3%); 49 were related to OTA (15.2%); 36 were related to ZEN (11.1%); 31 were related to FUM (9.6%); 23 were related to DON (7.1%); and 12 were related to T-2 toxin (3.7%) (Table 2).

Most studies were on finished feed (117/323, 36.2%) and cereals (115/323, 35.6%), followed by oil seed meal/cake (47/323, 14.6%), silage (10/323, 3.1%), wheat bran (9/323, 2.8%), hay (7/323, 2.2%), gluten meal (4/323, 1.2%), animal protein-based meal (4/323, 1.2%), straw (4/323, 1.2%), beet pulp (3/323, 0.9%), and dried bread (3/323, 0.9%) (Table 3).

**Figure 2:**
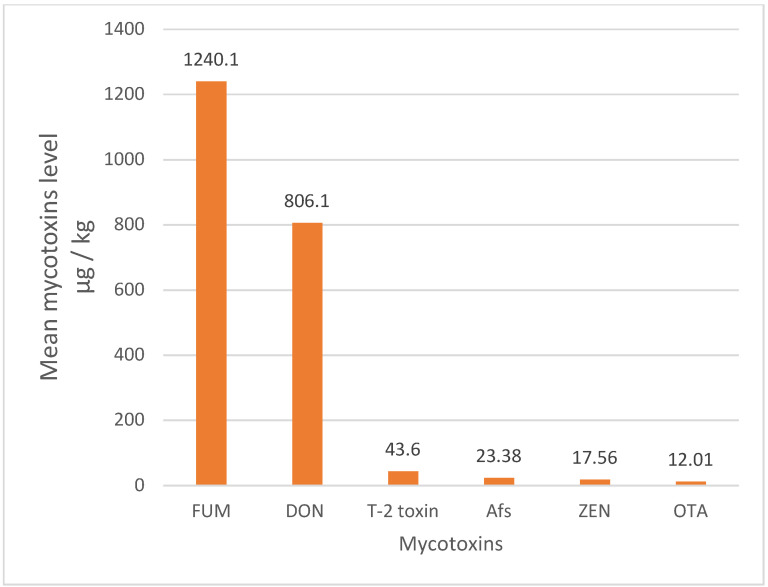
Mean level of mycotoxins in animal feed in the MENA region. Aflatoxins (AFs), deoxynivalenol (DON), zearalenone (ZEA), T-2 toxin, fumonisins (FUM), and ochratoxin A (OTA).

**Figure 3:**
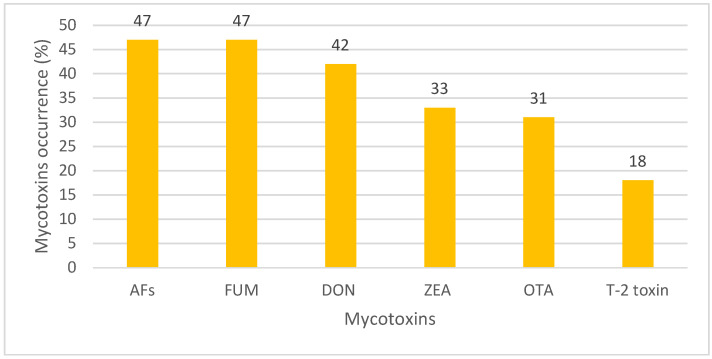
Occurrence of different mycotoxins in animal feed in the MENA region. Aflatoxins (AFs), deoxynivalenol (DON), zearalenone (ZEA), T-2 toxin, fumonisins (FUM), and ochratoxin A (OTA).

**Figure 4:**
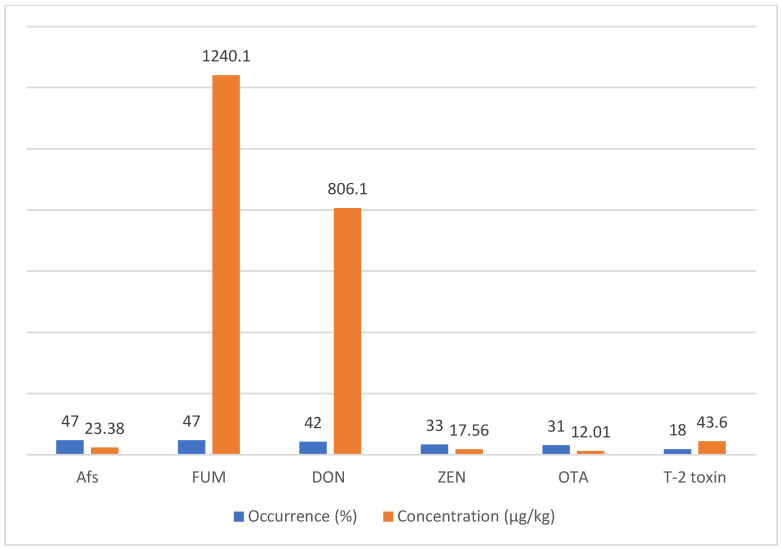
Occurrence and concentration of mycotoxins in animal feed in the MENA region. Aflatoxins (AFs), deoxynivalenol (DON), zearalenone (ZEA), T-2 toxin, fumonisins (FUM), and ochratoxin A (OTA).

The rank order of the mean mycotoxins level in animal feeds was as follows: FUM (1240.1 μg/kg), DON (806.1 μg/kg), T-2 toxin (43.60 μg/kg), AFs (23.38 μg/kg), ZEN (17.56 μg/kg), and OTA (12.01 μg/kg) (Table 4) (Figure 2). The overall occurrence of mycotoxins in animal feeds was 42%, with a remarkable heterogeneity (*I*^2^ = 96.71%, Cochrane Q test’s *p* < 0.001). Based on the mycotoxin type, the occurrence rank order was as follows: FUM (47%) ~ AFs (47%) > DON (42%) > ZEA (33%) > OTA (31%) > T-2 toxin (18%) (Figure 3). The occurrence and concentration of mycotoxins are shown in (Figure 4).

**Figure 5:**
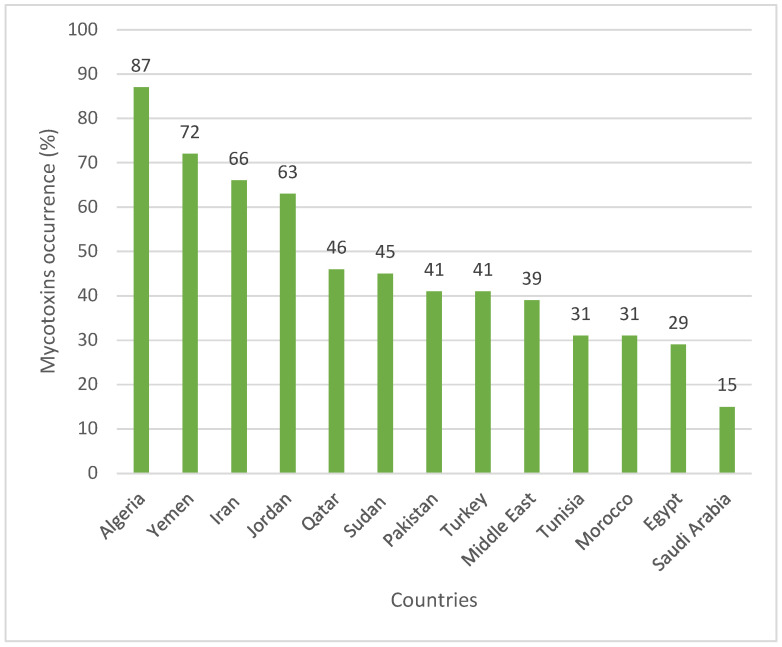
Occurrence of mycotoxins in animal feed in the countries of the MENA region.

Regarding the countries, the rank order of mycotoxins occurrence was Algeria (87%) > Yemen (72%) > Iran (66%) > Jordan (63%) > Qatar (46%) > Sudan (45%) > Pakistan (41%) ~ Turkey (41%) > Middle East (39%) > Tunisia (31%) ~ Morocco (31%) > Egypt (29%) > Saudi Arabia (15%) (Table 4) (Figure 5).

**Figure 6:**
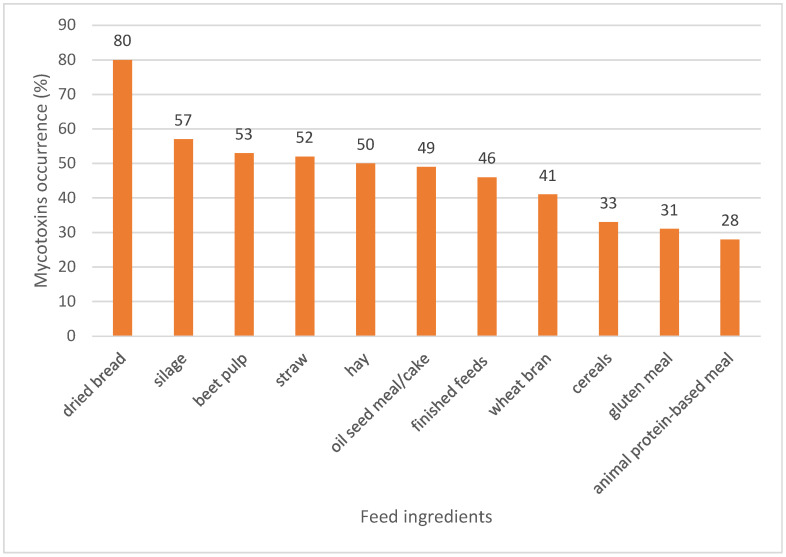
Occurrence of mycotoxins in different animal feed ingredients in the MENA region.

The occurrence of mycotoxins in finished feeds was 46%. Considering the feed ingredients, the lowest and highest mycotoxin occurrences were found in an animal protein-based meal (28%) and dried bread (80%), respectively. The mycotoxin occurrence in the other feed ingredients was ranked as follows: silage (57%) > beet pulp (53%) > straw (52%) > hay (50%) > oil seed meal/cake (49%) > wheat bran (41%) > cereals (33%) > gluten meal (31%) (Figure 6).

## 3. Discussion

In the present study, the highest occurrence of mycotoxins in animal feed in MENA was related to AFs and FUM (47%), and the lowest was related to T-2 toxin (18%). Additionally, the highest concentration of mycotoxin contamination in animal feed in the MENA region was related to FUM (1240.1 μg/kg) and then DON (806.1 μg/kg), and the lowest concentration was related to T-2 toxin (12.01 μg/kg). In most parts of the world, except North America, the highest concentrations of mycotoxin contamination in animal feed were related to FUM and then DON [38]. In a study of mycotoxin contamination in animal feed samples from a large geographical area in the Middle East and Africa, the highest incidence of mycotoxin contamination was related to FUM. A total of 83% of the samples were contaminated with FUM, and the average concentration was 713 μg/kg [82]. Additionally, in one study, contamination of Iranian dairy cows’ feed with AFB1, AFB2, AFG1, and AFG2 was 82.5%, 69.37%, 43.12%, and 41.87%, respectively [74]. On the other hand, Iran has one of the highest AFM1 contamination in ultra-high temperature processing (81%) of milk [55]. High contamination of AFM1 in milk can be due to the high level of AFB1 in animal feed, posing many risks to the human consumer community. One study found an association between AF exposure and the risk of liver cancer in humans [118]. A study of biomarkers of mycotoxin exposure in human urine has shown that Asian and African countries have the highest food exposure to AFs, FUM, ZEN, and DON [119].

Several factors can affect mycotoxins’ occurrence and concentration, such as climatic conditions (humidity, temperature, and precipitation), geographical location, and drought [120]. Mycotoxin production by mycotoxin-producing fungi is a complex and multifactorial process mainly dependent on establishing favorable environmental conditions for the growth of fungi. Contamination of animal feed with mycotoxins is predicted to affect climatic conditions [14] significantly. Climate is the most important factor in the occurrence of mycotoxins in animal feed, so in Europe, rainfall can be an important risk factor for mycotoxins in animal feed [121].

MENA is one of the driest and most vulnerable regions in the world to climate change. Contrary to popular belief, water scarcity is not the leading cause of vulnerability in these countries [122]. Yemen is the most vulnerable country in the MENA region to climate change [122], which in the present study also has a very high occurrence of mycotoxins in feed. The 10-year change in climate change score by 2020 has been different for the countries in the present study. Algeria, which has the highest occurrence of mycotoxins in animal feed, has a worsening score of about 6 points over its 10-year climate change score and has the worst situation among MENA countries. The climate change score of Tunisia, Morocco, Egypt, and Saudi Arabia, which have the lowest mycotoxin occurrence in animal feed, has improved in 10 years better than the global average [123]. The present study’s findings strengthen the hypothesis of the importance of climate change in the occurrence of mycotoxin contamination in animal feed.

Crop variety, crop rotation, tillage, and planting date are among the causes that have been considered effective in the occurrence of mycotoxin contamination in the components of animal feed. For example, plowing, early planting of corn, and avoiding planting corn before wheat cultivation are some things that reduce the risk of mycotoxin contamination in animal feed [121]. Using fungicides and insect damage can also affect the concentration of mycotoxins produced [124,125]. There are conflicting results and opinions about the contamination of organic products with mycotoxins, and it cannot be stated with certainty that the occurrence of mycotoxin contamination in them is more or less due to the lack of pesticide use [121,126,127]. It seems that farmers’ knowledge about mycotoxin contaminants, as well as risk factors for fungal and mycotoxin contaminants, should be increased. However, in some studies, this awareness has yet to be useful even in a European country such as Italy [109,121]. Post-harvest stages of animal feed components, including drying, transport, and storage, are the most critical stages in which mycotoxin contamination can occur [128].

Many MENA countries import food and feed, and contamination of feed with mycotoxins can occur before importation, transport, or storage until use [129]. Economic sanctions on Iran have been a significant obstacle to the Clean Development Mechanism (CDM) protocol [123]. Sanctions have prevented Iran from accessing clean technology for environmentally friendly development in polluting industries such as petrochemicals, refineries, smelters, and automobiles. Interestingly, out of 22 registered CDM projects, only one has received certification. All of these can effectively worsen the climate change situation, consequently increasing the occurrence of mycotoxin contamination in Iran’s livestock feed. Pakistan, which borders Iran, has about 68% more CDM projects than Iran, and mycotoxin contamination occurrence in Pakistan is 25% lower than in Iran. Yemen, where no CDM project has been registered, is worse off than Iran and ranks second in MENA’s mycotoxin contamination occurrence of animal feed. Sanctions affect countries’ access to global feed trade [130], which can lead farmers to turn to food waste in animal feed. As the present study showed, most mycotoxin contamination was in food waste such as dry bread, and the reckless introduction of these substances into animal feed can increase the occurrence of mycotoxin contamination.

The focus of digital agricultural development in MENA is on economic goals, and social and environmental challenges play a minor role in using digital technologies in the agricultural sector [122]. However, in developing countries such as Sudan and war-torn countries such as Yemen, there needs to be more knowledge to develop new technologies in agriculture and animal husbandry [122]. Thus, the lack of knowledge in animal feed production, processing, transportation, and consumption has led to a high occurrence of mycotoxin contamination in Yemen.

The results of the present study showed that the highest occurrence of mycotoxins in the MENA region was in dried bread, with 80%, and the lowest incidence of mycotoxins was related to an animal protein-based meal, with 28%. In the Messripour study, the highest incidence of AFs in animal feed was related to dry bread, 64% of which was contaminated with AFB1. The cause of this high contamination in dry bread has been attributed to improper storage. [131]. Despite this high contamination with mycotoxins in recycled bread, many farmers in MENA are forced to use these resources for animal feed [65,73,83,86,92]. Countries have to make optimal use of resources to provide animal feed. About 5 million tons of bread, pastry, and cereals are turned into animal feed in Europe [132]. It is estimated that by 2050 the demand for animal protein will increase by 70%. At the same time, UN member states are committed to reducing food waste by 50% by 2030 [132]. Therefore, it is necessary to use human food waste efficiently, and one of the easiest things to do is to use these human nutritional wastes in livestock feed. However, the use of these food wastes may involve chemical hazards (mycotoxins, antibiotics, heavy metals, gossypol, pesticides, dioxins, and biogenic amines), biological hazards (bacterial, fungal, parasitic, viral, and prion), and physical hazards (metal, glass, plastic, and other) for animals. Even the consumption of the products of these animals is harmful to human health [133,134]. Food waste may account for up to 65% of the dry matter consumed by livestock [135]. Given the high incidence of mycotoxin contamination in them, the use of these wastes is a significant risk factor for mycotoxins entering the feed.

The second rank of mycotoxin contamination occurrence is related to silage. Silage, especially corn silage, is an important component of dairy cows’ diets that can be contaminated with mycotoxins before harvest, during transportation, storage, after silage, or during use. Proper management of the silage to prevent fungal growth before and after silage and using chemical or biological additives can minimize the mycotoxin contamination of the silage [124,136]. However, due to the time between the harvest, processing, and consumption of silage, there is likely to be higher mycotoxin contamination. In one study, total DON and ZEA intake by dairy cows from silage were 3.5 and 2.9 times higher than compound feed, respectively [137]. In the present study, mycotoxin contamination in silage, straw, and hay was estimated to be more than 50%. Because most of the dry matter intake of ruminants is related to the forage part of the diet, mycotoxin contamination of forage is probably the cause of the most mycotoxins received by ruminants [38].

The chemical and physical properties of animal feed components can also affect the occurrence and concentration of mycotoxins, such as temperature, water content, pH, enzymatic activity, micronutrients, and macronutrients [54]. Various raw material processing methods can reduce the contamination of mycotoxins, such as FUM [54], which is why if the final feed is appropriately prepared and stored, it will be less contaminated than the raw components.

Different physical, chemical, and biological methods are used to reduce mycotoxin. Methods such as pulsed electric field process, oscillating magnetic field, high-pressure homogenization, X-ray methods, membrane filtration, and photolytic and photocatalytic methods have been used as non-thermal methods to reduce mycotoxins in food and feed [138]. Essential oils and plant extracts can decrease mycotoxin synthesis and change mycotoxins’ normal structure [139]. Nanoformulations of phytochemicals can be fungicides and detoxify the mycotoxins [140]. Recombinant mycotoxin-degrading enzymes can use for the detoxification of mycotoxins [141]. A microbial complex can simultaneously degrade different mycotoxins [141]. Adding probiotics and yeast to animal feed can reduce mycotoxins’ concentration or adverse effects and improve feed conversion ratio, immune status, and enzyme activity [142,143,144,145,146].

Due to the high incidence of mycotoxin contamination in animal feed in the MENA region and, on the other hand, the limitations and time-consuming implementation of macro-strategies to combat mycotoxins, more straightforward strategies to reduce mycotoxin contamination in animal feed should be used as an urgent solution. This solution may include using mycotoxin to inactivate, absorb, or degrade feed additives [121].

Studies have shown that ruminal fluid is one of the best binders for AFs. However, different conditions, such as pH, temperature, and type of AFs, can affect this ability [147]. Some research results have shown that adding some animal feed additives, such as *Solis Mos*, can reduce the transmission of feed AFB1 to milk as AFM1 [148]. Adding a toxin binder such as *Mycofix* to DON- and FUM-contaminated TMR feed can prevent the adverse effects of mycotoxins on milk, diet, immunity, and metabolic profile in dairy cows [24]. Additionally, anti-mycotoxin feed additives in broiler diets can reduce the biochemical changes caused by mycotoxins [26].

Diet composition can also affect the effect of mycotoxins on animal health and productivity. A study showed that higher levels of protein and methionine could reduce the adverse effect of mycotoxin on pig weight gain [25].

## 4. Conclusions

According to the results of the present study, 46% of MENA’s animal feed was contaminated with mycotoxins, which is a danger to the health of animals and humans consuming animal products in this region. The highest contamination percentage was related to FUM, AFs, and DON mycotoxins, and FUM and DON had the highest concentration of mycotoxin contamination. The dominant mycotoxins in the MENA are dangerous, and mycotoxins such as AFs can enter milk and dairy products and endanger human health. Algeria, Yemen, and Iran were, respectively, the most contaminated countries in terms of mycotoxin contamination of animal feed. These countries are not in a good situation dealing with climate change, and Yemen and Iran are experiencing a bad economic situation due to sanctions. However, these countries’ agricultural and health authorities should have detailed control programs to monitor mycotoxin contamination in customs, processing, and animal feed consumption. Dry bread is a human food waste used as animal feed in MENA. In the present study, it was the most contaminated animal feed, followed by silage, beet pulp, and fodder, which were the most contaminated with mycotoxins. Poor livestock farmers being forced to misuse food waste in animal feed, such as dry bread, can cause mycotoxin contamination in animal feed. Education on the correct preparation and storage methods of silage and fodder can effectively reduce the incidence of mycotoxin contamination in these materials. Additionally, different toxin binders, such as probiotics, were used to reduce the load of mycotoxin contamination in livestock feed.

Co-occurrence of mycotoxins in animal feed can have different effects on animals, most of which are synergistic and can pose a greater risk to animal and even human health [57]. Studies have shown that 30 to 100% of animal feed samples are contaminated with two or more mycotoxins, and this type of contamination is higher in Asia than in Europe and America. [38]. Therefore, permanent laboratory or field strategies should be considered to monitor mycotoxins’ co-occurrence in animal feed. The occurrence of mycotoxins such as nivalenol, citrinin, beauvericin, moniliformin, or enniatins should also be evaluated in future studies. Rapid screening with accurate methods to identify mycotoxins in animal feed is crucial to prevent the spread of contamination or the arrival of contaminated animal feed for animal consumption.

## 5. Materials and Methods

**Figure 7:**
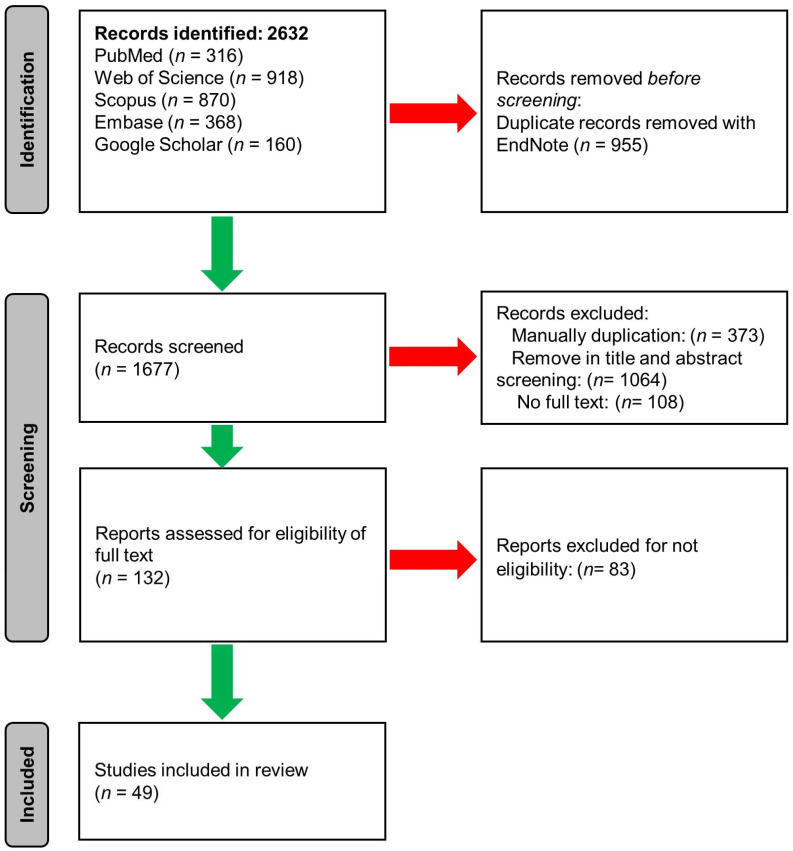
Systematic review PRISMA flow diagram.

The present systematic review was conducted based on the Cochrane protocol according to the PRISMA guidelines (Figure 7). The search strategy was performed from 2010 to 2020 in international databases such as PubMed, Scopus, Web of Science, Embase, and Google Scholar (as gray literature). Search terms used were: (“mycotoxin” OR “aflatoxin” OR “ochratoxin” OR “trichothecene” OR “deoxynivalenol” OR “zearalenone” OR “fumonisin”) AND (“iran” OR “iraq” OR “qatar” OR “turkey” OR “saudi” OR “afghanistan” OR “pakistan” OR “algeria” OR “armenia” OR “azerbayjan” OR “bahrain” OR “cyprus” OR “djibouti” OR “egypt” OR “georgia” OR “israel” OR “jordan” OR “kuwait” OR “lebanon” OR “libya” OR “malta” OR “mauritania” OR “morocco” OR “oman” OR “palestine” OR “somalia” OR “sudan” OR “syria” OR “tunisia” OR “emirates” OR “western”sahra” OR “yemen”) AND (“feed” OR “feedstuff” OR “maize” OR “fodder” OR “forage” OR “hay” OR “straw” OR “silage” OR “soy” OR “soybean” OR “meal” OR “wheat” OR “barley” OR “rice” OR “corn” OR “sorghum” OR “palm” OR “cottonseed” OR “sunflower”). Searches were performed on titles, abstracts, and keywords of articles. Duplicate articles were removed using EndNote X8 (Thomson Reuters, Toronto, Canada). A manual search in the article references section was performed to prevent missing relevant studies. Two independent reviewers screened studies’ abstracts and full texts based on inclusion criteria. After deleting several articles by screening the title and abstract, the full text of the articles was first searched and added by EndNote software. Using the DOI and other article specifications and Urmia University’s access to scientific databases, it was searched and added to the EndNote library. Original articles that reported the concentrations and occurrence of mycotoxins in animal feed in the MENA region and published online between 2010 and 2022 were included in the study.

Criteria for eligibility of articles for inclusion in the study included: (a) the full text of the article is available in English; (b) concentration and/or occurrence of at least one of the mycotoxins in at least one of the types of animal feed be reported; (c) cross-sectional studies are carefully described; (d) accurate assessment methods are used; (e) data with effective size (concentration and/or prevalence) in animal feed are reported. In this regard, other ecological studies, genetic research, case reports, animal studies, dissertations, and review articles were excluded from the systematic review. Some unrelated articles were deleted in the first stage based on the title evaluation. In the second stage, the abstract of the articles was evaluated, and disproportionate articles were deleted; finally, the full text of related articles was searched and reviewed.

Data extraction was performed from eligible articles. Extracted data included first author name, sampling year(s), publication year, type of feed, type of mycotoxin, standard deviation (SD) or standard error (SE), mean or median level of mycotoxin, minimum and maximum level of mycotoxin, total number of samples, number of positive samples, continent, country, mycotoxin detection method and its limit of detection (LOD), and quantification (LOQ). All reported units for mycotoxin levels were converted to µg/kg. Converting SD to SE and vice versa was conducted using the following formula (Equation (1)):

(1) $$\mathrm{SE}=\frac{\mathrm{SD}}{\sqrt{\mathrm{sample}\;\mathrm{size}}}$$

Data meta-analysis was performed using Stata 14.0 (Statistical Software, College Station, TX, USA, 2015). The relationship between the total number of samples (ni) and positive samples (pi) was the prevalence (*p* = pi/ni), which was defined as the effect size (ES) and expressed as a percentage. The Metaprop command calculated the prevalence of mycotoxins. A 95% confidence interval (CI) was obtained for the articles. The combined concentrations of mycotoxins were estimated using SE (standard error) and mean. The weight of each study (*W_i_*) was determined according to each study’s accuracy, which was inversely related to SE (Equation (2)). A meta-analysis of the prevalence and concentration of mycotoxins was performed using a weighted average (combined average). The average weight (AW) was calculated according to the accuracy of each study, which is correlated with standard error (Equation (3)). ∑*W* in the third equation shows the sum of *W_i_*.

In articles that did not mention SE, the number of samples was calculated using SD. The random-effects model was used to estimate the overall effect because the eligible studies were performed in various settings. The Cochrane Q test and *I*^2^ index evaluated the heterogeneity among the studies. The Cochrane Q test’s *p* ≤ 0.050 indicates the presence of heterogeneity, and *I*^2^ > 50% indicates the high heterogeneity among the studies. When the *I*^2^ index is less than 50%, the heterogeneity is low, but in the present study, the *I*^2^ index was higher than 50%, so the random effect model ^3^ was used to evaluate the combined ES. Due to severe data heterogeneity, the instantaneous model performed meta-regression between studies. The difference was statistically significant when *p* < 0.001. Finally, tables and graphs of the results were prepared [67]. The formulas used are as follows:

(2) $$W_{i}=\frac{1}{{\mathrm{SE}}^{2}}$$

(3) $$AW=\frac{W_{i}}{\sum W}\times 100$$

## Figures and Tables

**Table 1:** Meta-analysis of the occurrence of mycotoxins in animal feed in the countries of the MENA region.

Country	No. of Trials	ES (95% CI)	*p*	*I*^2^ (%)	*P*_Q_
Pakistan	116	0.41 (0.35, 0.46)	<0.001	92.30	<0.001
Iran	52	0.66 (0.56, 0.76)	<0.001	96.05	<0.001
Turkey	48	0.41 (0.31, 0.52)	<0.001	97.77	<0.001
Egypt	31	0.29 (0.18, 0.40)	<0.001	97.60	<0.001
Tunisia	21	0.31 (0.16, 0.47)	<0.001	97.36	<0.001
Qatar	18	0.46 (0.28, 0.64)	<0.001	78.24	<0.001
Saudi Arabia	11	0.15 (0.09, 0.23)	<0.001	93.53	<0.001
Middle East	16	0.39 (0.24, 0.56)	<0.001	95.9	–
Jordan	3	0.63 (0.25, 0.94)	<0.001	–	–
Sudan	3	0.45 (0.32, 0.59)	<0.001	–	–
Algeria	2	0.87 (0.74, 0.96)	<0.001	–	–
Morocco	1	0.31 (0.21, 0.43)	<0.001	–	–
Yemen	1	0.72 (0.60, 0.82)	<0.001	–	–
Overall estimate	323	0.42 (0.38, 0.46)	<0.001	96.71	<0.001

**Table 2:** Meta-analysis of the occurrence of mycotoxins in animal feed in the MENA region.

Mycotoxin Type	No. of Trials	ES (95% CI)	*p*	*I*^2^ (%)	*P*_Q_
Aflatoxins	172	0.47 (0.40, 0.53)	<0.001	97.35	<0.001
Ochratoxin A	49	0.31 (0.23, 0.39)	<0.001	91.34	<0.001
Zearalenone	36	0.33 (0.23, 0.43)	<0.001	93.98	<0.001
Fumonisins	31	0.47 (0.35, 0.60)	<0.001	96.79	<0.001
Deoxynivalenol	23	0.42 (0.32, 0.53)	<0.001	92.42	<0.001
T-2 toxin	12	0.18 (0.06, 0.34)	<0.001	96.97	<0.001
Overall estimate	323	0.42 (0.38, 0.46)	<0.001	96.71	<0.001

**Table 3:** Meta-analysis of the occurrence of mycotoxins in animal feed ingredients in the MENA region.

Ingredients	No. of Trials	ES (95% CI)	*p*	*I*^2^ (%)	*P*_Q_
Finished feed	117	0.46 (0.39, 0.54	<0.001	97.94	<0.001
Cereals	115	0.33 (0.28, 0.37)	<0.001	93.33	<0.001
Oilseed meal/cake	47	0.49 (0.40, 0.58)	<0.001	91.68	<0.001
Silage	10	0.57 (0.33, 0.80)	<0.001	96.01	<0.001
Wheat bran	9	0.41 (0.14, 0.72)	<0.001	95.46	<0.001
Hay	7	0.50 (0.26, 0.74)	<0.001	93.87	<0.001
Gluten meal	4	0.31 (0.17, 0.46)	<0.001	36.13	0.20
Animal protein-based meal	4	0.28 (0.05, 0.58)	<0.001	78.84	<0.001
Straw	4	0.52 (0.27, 0.76)	<0.001	90.71	<0.001
Beet pulp	3	0.53 (0.22, 0.83)	<0.001	–	–
Dried bread	3	0.80 (0.61, 0.94)	<0.001	–	–
Overall estimate	323	0.42 (0.38, 0.46)	<0.001	96.71	<0.001

**Table 4:** Meta-analysis of concentrations of mycotoxin types in animal feed in the MENA region.

Mycotoxin Type	No. of trials	ES (95% CI)	*p*	*I*^2^ (%)	*P*_Q_
Aflatoxins	114	23.38 (−47.98, 94.74)	0.521	100	<0.001
Ochratoxin A	43	12.01 (10.93, 13.08)	<0.001	99.1	<0.001
Zearalenone	11	17.56 (15.07, 20.05)	<0.001	99.8	<0.001
Fumonisins	6	1240.1 (841.9, 1638.3)	<0.001	100	<0.001
Deoxynivalenol	5	806.1 (1.03, 2615.8)	0.038	100	<0.001
T-2 toxin	7	43.60 (−28.63, 115.8)	0.237	100	<0.001

## Data Availability

Not applicable.

## Supplementary Materials

The following supporting information can be downloaded at: https://www.mdpi.com/article/10.3390/toxins15030214/s1; Table S1: Details of data extracted from selected studies that met the inclusion criteria.
