# Supplementary material for: Prevalence and Concentration of Mycotoxins in Animal Feed in the Middle East and North Africa (MENA): A Systematic Review and Meta-Analysis
Source: Toxins (Basel). 2023 Mar 10;15(3):214. doi: 10.3390/toxins15030214 (PMC10054064; doi:10.3390/toxins15030214)
Supplement: Supplementary file 1 [file toxins-15-00214-s001.zip › toxins-2091322-supplementary.pdf]

Table S1: Details of data extracted from selected studies that met the inclusion criteria.

First author	Sampling	Publish Year	Type of cereals	Type of Mycotoxin	SD	SE	Mean or median* of Mycotoxin level (µg/kg)	Minimum of Mycotoxin level (µg/kg)	Maximum of Mycotoxin level (µg/kg)	Total Number samples	Number positive samples	Incidence (%)	Continent	Country	LOD (µg/kg)	LOQ (µg/kg)	Method	Full Citation
Demir		2010	Fresh Corn	Fumonisin B1			1330	50	14280	71	34	48	Asia	Turkey	20	40	HPLC	DEMIR, C., SIMSEK, O. & ARICI, M. 2010. Incidence of Fusarium verticillioides and Levels of Fumonisin B-1 and B-2 in Corn in Turkey. Food Science and Biotechnology, 19, 1103-1106.
Demir		2010	Fresh Corn	Fumonisin B2			830	50	4200	71	14	20	Asia	Turkey	30	60	HPLC	DEMIR, C., SIMSEK, O. & ARICI, M. 2010. Incidence of Fusarium verticillioides and Levels of Fumonisin B-1 and B-2 in Corn in Turkey. Food Science and Biotechnology, 19, 1103-1106.
Demir		2010	Fresh Corn	Fumonisins			1670	50	17130	71	34	48	Asia	Turkey			HPLC	DEMIR, C., SIMSEK, O. & ARICI, M. 2010. Incidence of Fusarium verticillioides and Levels of Fumonisin B-1 and B-2 in Corn in Turkey. Food Science and Biotechnology, 19, 1103-1106.
Demir		2010	Stored Corn	Fumonisin B1			3760	50	25170	29	18	62	Asia	Turkey	20	40	HPLC	DEMIR, C., SIMSEK, O. & ARICI, M. 2010. Incidence of Fusarium verticillioides and Levels of Fumonisin B-1 and B-2 in Corn in Turkey. Food Science and Biotechnology, 19, 1103-1106.
Demir		2010	Stored Corn	Fumonisin B2			1550	50	5710	29	11	38	Asia	Turkey	30	60	HPLC	DEMIR, C., SIMSEK, O. & ARICI, M. 2010. Incidence of Fusarium verticillioides and Levels of Fumonisin B-1 and B-2 in Corn in Turkey. Food Science and Biotechnology, 19, 1103-1106.
Demir		2010	Stored Corn	Fumonisins			4710	50	31430	29	18	62	Asia	Turkey			HPLC	DEMIR, C., SIMSEK, O. & ARICI, M. 2010. Incidence of Fusarium verticillioides and Levels of Fumonisin B-1 and B-2 in Corn in Turkey. Food Science and Biotechnology, 19, 1103-1106.
Karami	2004-2005	2010	corn	Deoxynivalenol			116	54	518	60	46	76	Asia	Iran	10		HPLC	Karami-Osboo, R., Mirabolfathy, M., & Aliakbari, F. (2010). Natural Deoxynivalenol Contamination of Corn Produced in Golestan and Moqan Areas in Iran. Journal of Agricultural Science and Technology, 12(2), 233-239.
Anjum		2011	Wheat	Ochratoxin A	111.600	22.320	59.43	25	98	25	7	28	Asia	Pakistan			TLC	Anjum, M. A., Sahota, A. W., Akram, M., & Ali, I. (2011). Prevalence of Mycotoxins in Poultry Feeds and Feed Ingriedents in Punjab (Pakistan). Journal of Animal and Plant Sciences, 21(2), 117-120.
Anjum		2011	Maize	Ochratoxin A	113.450	22.690	111.2	75	140	25	10	40	Asia	Pakistan			TLC	Anjum, M. A., Sahota, A. W., Akram, M., & Ali, I. (2011). Prevalence of Mycotoxins in Poultry Feeds and Feed Ingriedents in Punjab (Pakistan). Journal of Animal and Plant Sciences, 21(2), 117-120.
Anjum		2011	Rice	Ochratoxin A	48.250	9.650	27.4	15	40	25	5	20	Asia	Pakistan			TLC	Anjum, M. A., Sahota, A. W., Akram, M., & Ali, I. (2011). Prevalence of Mycotoxins in Poultry Feeds and Feed Ingriedents in Punjab (Pakistan). Journal of Animal and Plant Sciences, 21(2), 117-120.

Anjum	2011	Sorghum	Ochratoxin A	27.300	5.460	33.6	25	40	25	5	20	Asia	Pakistan	TLC	Anjum, M. A., Sahota, A. W., Akram, M., & Ali, I. (2011). Prevalence of Mycotoxins in Poultry Feeds and Feed Ingriedents in Punjab (Pakistan). Journal of Animal and Plant Sciences, 21(2), 117-120.
Anjum	2011	Barley	Ochratoxin A	43.850	8.770	30.33	15	112	25	6	24	Asia	Pakistan	TLC	Anjum, M. A., Sahota, A. W., Akram, M., & Ali, I. (2011). Prevalence of Mycotoxins in Poultry Feeds and Feed Ingriedents in Punjab (Pakistan). Journal of Animal and Plant Sciences, 21(2), 117-120.
Anjum	2011	Peanut meal	Ochratoxin A	51.150	10.230	49.2	30	58	25	5	20	Asia	Pakistan	TLC	Anjum, M. A., Sahota, A. W., Akram, M., & Ali, I. (2011). Prevalence of Mycotoxins in Poultry Feeds and Feed Ingriedents in Punjab (Pakistan). Journal of Animal and Plant Sciences, 21(2), 117-120.
Anjum	2011	Millet	Ochratoxin A			5	5	8	25	1	4	Asia	Pakistan	TLC	Anjum, M. A., Sahota, A. W., Akram, M., & Ali, I. (2011). Prevalence of Mycotoxins in Poultry Feeds and Feed Ingriedents in Punjab (Pakistan). Journal of Animal and Plant Sciences, 21(2), 117-120.
Anjum	2011	Grams	Ochratoxin A	10.250	2.050	12.67	10	15	25	3	12	Asia	Pakistan	TLC	Anjum, M. A., Sahota, A. W., Akram, M., & Ali, I. (2011). Prevalence of Mycotoxins in Poultry Feeds and Feed Ingriedents in Punjab (Pakistan). Journal of Animal and Plant Sciences, 21(2), 117-120.
Anjum	2011	Cotton Seed meal	Ochratoxin A	41.054	9.180	32.33	20	42	20	3	15	Asia	Pakistan	TLC	Anjum, M. A., Sahota, A. W., Akram, M., & Ali, I. (2011). Prevalence of Mycotoxins in Poultry Feeds and Feed Ingriedents in Punjab (Pakistan). Journal of Animal and Plant Sciences, 21(2), 117-120.
Anjum	2011	Soybean meal	Ochratoxin A	38.908	8.700	22.75	28	33	20	4	20	Asia	Pakistan	TLC	Anjum, M. A., Sahota, A. W., Akram, M., & Ali, I. (2011). Prevalence of Mycotoxins in Poultry Feeds and Feed Ingriedents in Punjab (Pakistan). Journal of Animal and Plant Sciences, 21(2), 117-120.
Anjum	2011	Rape seed meal	Ochratoxin A	16.726	3.740	33	30	37	20	3	15	Asia	Pakistan	TLC	Anjum, M. A., Sahota, A. W., Akram, M., & Ali, I. (2011). Prevalence of Mycotoxins in Poultry Feeds and Feed Ingriedents in Punjab (Pakistan). Journal of Animal and Plant Sciences, 21(2), 117-120.
Anjum	2011	Sun-flower meal	Ochratoxin A	61.671	13.790	50.33	15	68	20	6	30	Asia	Pakistan	TLC	Anjum, M. A., Sahota, A. W., Akram, M., & Ali, I. (2011). Prevalence of Mycotoxins in Poultry Feeds and Feed Ingriedents in Punjab (Pakistan). Journal of Animal and Plant Sciences, 21(2), 117-120.
Anjum	2011	Guar meal	Ochratoxin A	24.150	5.400	21.75	20	30	20	4	20	Asia	Pakistan	TLC	Anjum, M. A., Sahota, A. W., Akram, M., & Ali, I. (2011). Prevalence of Mycotoxins in Poultry Feeds and Feed Ingriedents in Punjab (Pakistan). Journal of Animal and Plant Sciences, 21(2), 117-120.
Anjum	2011	Corn gluten meal (60%)	Ochratoxin A	45.795	10.240	32.3	20	48	20	5	25	Asia	Pakistan	TLC	Anjum, M. A., Sahota, A. W., Akram, M., & Ali, I. (2011). Prevalence of Mycotoxins in Poultry Feeds and Feed Ingriedents in Punjab (Pakistan). Journal of Animal and Plant Sciences, 21(2), 117-120.
Anjum	2011	Corn gluten meal (30%)	Ochratoxin A	69.005	15.430	36	25	56	20	4	20	Asia	Pakistan	TLC	Anjum, M. A., Sahota, A. W., Akram, M., & Ali, I. (2011). Prevalence of Mycotoxins in Poultry Feeds and Feed Ingriedents in Punjab (Pakistan). Journal of Animal and Plant Sciences, 21(2), 117-120.
Anjum	2011	Rice polishing	Ochratoxin A	67.350	15.060	39.14	5	57	20	4	20	Asia	Pakistan	TLC	Anjum, M. A., Sahota, A. W., Akram, M., & Ali, I. (2011). Prevalence of Mycotoxins in Poultry Feeds

																		and Feed Ingriedents in Punjab (Pakistan). Journal of Animal and Plant Sciences, 21(2), 117-120.
Anjum		2011	Wheat bran	Ochratoxin A	27.906	6.240	11.67	12	10	20	3	15	Asia	Pakistan			TLC	Anjum, M. A., Sahota, A. W., Akram, M., & Ali, I. (2011). Prevalence of Mycotoxins in Poultry Feeds and Feed Ingriedents in Punjab (Pakistan). Journal of Animal and Plant Sciences, 21(2), 117-120.
Anjum		2011	Fish meal	Ochratoxin A	15.205	3.400	16.67	12	10	20	3	15	Asia	Pakistan			TLC	Anjum, M. A., Sahota, A. W., Akram, M., & Ali, I. (2011). Prevalence of Mycotoxins in Poultry Feeds and Feed Ingriedents in Punjab (Pakistan). Journal of Animal and Plant Sciences, 21(2), 117-120.
Anjum		2011	Blood meal	Ochratoxin A	17.889	4.000	14	12	10	20	3	15	Asia	Pakistan			TLC	Anjum, M. A., Sahota, A. W., Akram, M., & Ali, I. (2011). Prevalence of Mycotoxins in Poultry Feeds and Feed Ingriedents in Punjab (Pakistan). Journal of Animal and Plant Sciences, 21(2), 117-120.
Anjum		2011	Meat meal	Ochratoxin A	22.361	5.000	10	12	10	20	3	15	Asia	Pakistan			TLC	Anjum, M. A., Sahota, A. W., Akram, M., & Ali, I. (2011). Prevalence of Mycotoxins in Poultry Feeds and Feed Ingriedents in Punjab (Pakistan). Journal of Animal and Plant Sciences, 21(2), 117-120.
Ghiasian	2000	2011	Maize	Total Aflatoxins			2.92		2.9	15	1	6.7	Asia	Iran	0.1		HPLC	Ghiasian, S. A., Shephard, G. S., & Yazdanpanah, H. (2011). Natural occurrence of aflatoxins from maize in Iran. Mycopathologia, 172(2), 153-160. doi:10.1007/s11046-011-9405-y
Ghiasian	2000	2011	Maize	Total Aflatoxins	117.000	33.775	52.6		316.9	12	7	58.3	Asia	Iran	0.1		HPLC	Ghiasian, S. A., Shephard, G. S., & Yazdanpanah, H. (2011). Natural occurrence of aflatoxins from maize in Iran. Mycopathologia, 172(2), 153-160. doi:10.1007/s11046-011-9405-y
Ghiasian	2000	2011	Maize	Total Aflatoxins	8.730	2.333	6.52		12.7	14	2	14.3	Asia	Iran	0.1		HPLC	Ghiasian, S. A., Shephard, G. S., & Yazdanpanah, H. (2011). Natural occurrence of aflatoxins from maize in Iran. Mycopathologia, 172(2), 153-160. doi:10.1007/s11046-011-9405-y
Ghiasian	2000	2011	Maize	Total Aflatoxins	4.750	1.502	5.67		11.2	10	8	80	Asia	Iran	0.1		HPLC	Ghiasian, S. A., Shephard, G. S., & Yazdanpanah, H. (2011). Natural occurrence of aflatoxins from maize in Iran. Mycopathologia, 172(2), 153-160. doi:10.1007/s11046-011-9405-y
Ghiasian	2000	2011	Maize	Total Aflatoxins					316.9	51	17	33	Asia	Iran	0.1		HPLC	Ghiasian, S. A., Shephard, G. S., & Yazdanpanah, H. (2011). Natural occurrence of aflatoxins from maize in Iran. Mycopathologia, 172(2), 153-160. doi:10.1007/s11046-011-9405-y
Rodrigues	2009	2011	Maize	Fumonisins			987		4398	63	53	84	Asia/Africa	Middle East and Africa	25	80	HPLC	Rodrigues, I., Handl, J., & Binder, E. M. (2011). Mycotoxin occurrence in commodities, feeds and feed ingredients sourced in the Middle East and Africa. Food Addit Contam Part B Surveill, 4(3), 168-179. doi:10.1080/19393210.2011.589034
Rodrigues	2009	2011	Maize	Zearalenone			17		310	63	10	15	Asia/Africa	Middle East and Africa	10	25	HPLC	Rodrigues, I., Handl, J., & Binder, E. M. (2011). Mycotoxin occurrence in commodities, feeds and feed ingredients sourced in the Middle East and Africa. Food Addit Contam Part B Surveill, 4(3), 168-179. doi:10.1080/19393210.2011.589034
Rodrigues	2009	2011	Maize	Total Aflatoxins			28		343	63	22	34	Asia/Africa	Middle East	0.3	0.8	HPLC	Rodrigues, I., Handl, J., & Binder, E. M. (2011). Mycotoxin occurrence in commodities, feeds and feed ingredients sourced in the Middle East and

											and Africa				Africa. Food Addit Contam Part B Surveill, 4(3), 168-179. doi:10.1080/19393210.2011.589034
Rodrigues	2009	2011	Wheat/wheat bran	Fumonisins	29	404	32	3	9	Asia/Africa	Middle East and Africa	25	80	HPLC	Rodrigues, I., Handl, J., & Binder, E. M. (2011). Mycotoxin occurrence in commodities, feeds and feed ingredients sourced in the Middle East and Africa. Food Addit Contam Part B Surveill, 4(3), 168-179. doi:10.1080/19393210.2011.589034
Rodrigues	2009	2011	Wheat/wheat bran	Zearalenone	31	392	32	8	25	Asia/Africa	Middle East and Africa	10	25	HPLC	Rodrigues, I., Handl, J., & Binder, E. M. (2011). Mycotoxin occurrence in commodities, feeds and feed ingredients sourced in the Middle East and Africa. Food Addit Contam Part B Surveill, 4(3), 168-179. doi:10.1080/19393210.2011.589034
Rodrigues	2009	2011	Wheat/wheat bran	Total Aflatoxins	1	7	32	6	18	Asia/Africa	Middle East and Africa	0.3	0.8	HPLC	Rodrigues, I., Handl, J., & Binder, E. M. (2011). Mycotoxin occurrence in commodities, feeds and feed ingredients sourced in the Middle East and Africa. Food Addit Contam Part B Surveill, 4(3), 168-179. doi:10.1080/19393210.2011.589034
Rodrigues	2009	2011	Finished feed	Fumonisins	713	2588	119	99	83	Asia/Africa	Middle East and Africa	25	80	HPLC	Rodrigues, I., Handl, J., & Binder, E. M. (2011). Mycotoxin occurrence in commodities, feeds and feed ingredients sourced in the Middle East and Africa. Food Addit Contam Part B Surveill, 4(3), 168-179. doi:10.1080/19393210.2011.589034
Rodrigues	2009	2011	Finished feed	Zearalenone	14	138	119	33	27	Asia/Africa	Middle East and Africa	10	25	HPLC	Rodrigues, I., Handl, J., & Binder, E. M. (2011). Mycotoxin occurrence in commodities, feeds and feed ingredients sourced in the Middle East and Africa. Food Addit Contam Part B Surveill, 4(3), 168-179. doi:10.1080/19393210.2011.589034
Rodrigues	2009	2011	Finished feed	Total Aflatoxins	24	213	119	50	42	Asia/Africa	Middle East and Africa	0.3	0.8	HPLC	Rodrigues, I., Handl, J., & Binder, E. M. (2011). Mycotoxin occurrence in commodities, feeds and feed ingredients sourced in the Middle East and Africa. Food Addit Contam Part B Surveill, 4(3), 168-179. doi:10.1080/19393210.2011.589034
Rodrigues	2009	2011	Soybean/Soybean meal	Fumonisins	71	2035	33	4	12	Asia/Africa	Middle East and Africa	25	80	HPLC	Rodrigues, I., Handl, J., & Binder, E. M. (2011). Mycotoxin occurrence in commodities, feeds and feed ingredients sourced in the Middle East and Africa. Food Addit Contam Part B Surveill, 4(3), 168-179. doi:10.1080/19393210.2011.589034
Rodrigues	2009	2011	Soybean/Soybean meal	Zearalenone	0	0	33	0	0	Asia/Africa	Middle East and Africa	10	25	HPLC	Rodrigues, I., Handl, J., & Binder, E. M. (2011). Mycotoxin occurrence in commodities, feeds and feed ingredients sourced in the Middle East and Africa. Food Addit Contam Part B Surveill, 4(3), 168-179. doi:10.1080/19393210.2011.589034
Rodrigues	2009	2011	Soybean/Soybean meal	Total Aflatoxins	4	42	33	8	24	Asia/Africa	Middle East and Africa	0.3	0.8	HPLC	Rodrigues, I., Handl, J., & Binder, E. M. (2011). Mycotoxin occurrence in commodities, feeds and feed ingredients sourced in the Middle East and Africa. Food Addit Contam Part B Surveill, 4(3), 168-179. doi:10.1080/19393210.2011.589034
Rodrigues	2009	2011	feed commodities	Fumonisins	528	10485	65	32	49	Asia/Africa	Middle East and Africa	25	80	HPLC	Rodrigues, I., Handl, J., & Binder, E. M. (2011). Mycotoxin occurrence in commodities, feeds and feed ingredients sourced in the Middle East and Africa

																		Africa. Food Addit Contam Part B Surveill, 4(3), 168-179. doi:10.1080/19393210.2011.589034
Rodrigues	2009	2011	feed commodities	Zearalenone			32		195	65	29	44	Asia/Africa	Middle East and Africa	10	25	HPLC	Rodrigues, I., Handl, J., & Binder, E. M. (2011). Mycotoxin occurrence in commodities, feeds and feed ingredients sourced in the Middle East and Africa. Food Addit Contam Part B Surveill, 4(3), 168-179. doi:10.1080/19393210.2011.589034
Rodrigues	2009	2011	feed commodities	Total Aflatoxins			57		556	65	59	90	Asia/Africa	Middle East and Africa	0.3	0.8	HPLC	Rodrigues, I., Handl, J., & Binder, E. M. (2011). Mycotoxin occurrence in commodities, feeds and feed ingredients sourced in the Middle East and Africa. Food Addit Contam Part B Surveill, 4(3), 168-179. doi:10.1080/19393210.2011.589034
Rodrigues	2009	2011	feed commodities	Ochratoxin A			14		31	11	10	90	Asia/Africa	Middle East and Africa	0.2	0.5	HPLC	Rodrigues, I., Handl, J., & Binder, E. M. (2011). Mycotoxin occurrence in commodities, feeds and feed ingredients sourced in the Middle East and Africa. Food Addit Contam Part B Surveill, 4(3), 168-179. doi:10.1080/19393210.2011.589034
Degirmencioglu	2007	2012	Layer feed	Total Aflatoxins	0.200	0.020	6.5	0.4	36.8	100	13	13	Asia	Turkey	1	2.5	ELISA	Degirmencioglu, N., Eseceli, H., Demir, E., & Senturklu, S. (2012). Evaluation of total aflatoxin, nitrate and nitrite levels in layer feed samples of companies producing their own feed in Edincik and Bandirma province of Turkey. Food Additives & Contaminants Part B-Surveillance, 5(2), 133-139. doi:10.1080/19393210.2012.680200
Degirmencioglu	2008	2012	Layer feed	Total Aflatoxins	0.200	0.020	7.3	0.5	47	100	15	15	Asia	Turkey	1	2.5	ELISA	Degirmencioglu, N., Eseceli, H., Demir, E., & Senturklu, S. (2012). Evaluation of total aflatoxin, nitrate and nitrite levels in layer feed samples of companies producing their own feed in Edincik and Bandirma province of Turkey. Food Additives & Contaminants Part B-Surveillance, 5(2), 133-139. doi:10.1080/19393210.2012.680200
Abbes	2008-2010	2012	Feed	AFB1	1.400	0.184	18.66	5.1	50.1	58	49	84	Africa	Tunisia	0.01		ELISA	Natural occurrence of aflatoxins (B-1 and M-1) in feed, plasma and raw milk of lactating dairy cows in Beja, Tunisia, using ELISA
Ali		2012	Animal feed	Aflatoxin B1			16.75		100	20	12	60	Africa	Sudan			HPLC	Ali, S. A., & Mohamed, A. A. (2012). Determination of Aflatoxins in Selected Foods and Animal Feed in Khartoum State, Sudan. I International Symposium on Mycotoxins in Nuts and Dried Fruits, 963, 231-235.
Ali		2012	Sorghum	Aflatoxin B1			6.95		125	35	15	42	Africa	Sudan			HPLC	Ali, S. A., & Mohamed, A. A. (2012). Determination of Aflatoxins in Selected Foods and Animal Feed in Khartoum State, Sudan. I International Symposium on Mycotoxins in Nuts and Dried Fruits, 963, 231-235.
Ali		2012	corn	Aflatoxin B1			1		5	20	7	35	Africa	Sudan			HPLC	Ali, S. A., & Mohamed, A. A. (2012). Determination of Aflatoxins in Selected Foods and Animal Feed in Khartoum State, Sudan. I International Symposium on Mycotoxins in Nuts and Dried Fruits, 963, 231-235.
Azizi	2009	2012	Concentrated feed	Aflatoxin B1						34	20	58	Asia	Iran	5		ELISA	Azizi, I. G., Ghadi, H., & Azarmi, M. (2012). Determination of Aflatoxin B-1 Levels of the

																Feedstuffs in Traditional and Semi-industrial Cattle Farms in Amol, Northern Iran. Asian Journal of Animal and Veterinary Advances, 7(6), 528-534. doi:10.3923/ajava.2012.528.534
Azizi	2009	2012	Beet pulp	Aflatoxin B1				32	14	43	Asia	Iran	5		ELISA	Azizi, I. G., Ghadi, H., & Azarmi, M. (2012). Determination of Aflatoxin B-1 Levels of the Feedstuffs in Traditional and Semi-industrial Cattle Farms in Amol, Northern Iran. Asian Journal of Animal and Veterinary Advances, 7(6), 528-534. doi:10.3923/ajava.2012.528.534
Azizi	2009	2012	Cotton meal	Aflatoxin B1				30	8	26	Asia	Iran	5		ELISA	Azizi, I. G., Ghadi, H., & Azarmi, M. (2012). Determination of Aflatoxin B-1 Levels of the Feedstuffs in Traditional and Semi-industrial Cattle Farms in Amol, Northern Iran. Asian Journal of Animal and Veterinary Advances, 7(6), 528-534. doi:10.3923/ajava.2012.528.534
Feizy		2012	Cotton seed	Total Aflatoxin	2.6		14.14	139	129	92	Asia	Iran	0.12	0.2	HPLC	Feizy, J., Beheshti, H. R., & Asadi, M. (2012). A survey of aflatoxin in cotton seed in Iran by HPLC with on-line photochemical derivatisation and fluorescence detection. Food Additives and Contaminants: Part B Surveillance, 5(3), 200-203. doi:10.1080/19393210.2012.696148
Khatoon	2007	2012	Maize	Aflatoxin B1	192	5	850	65	18	27.69	Asia	Pakistan		1	HPTLC	Khatoon, S., Hanif, N. Q., Tahira, I., Sultana, N., Sultana, K., & Ayub, N. (2012). Natural Occurrence of Aflatoxins, Zearalenone and Trichothecenes in Maize Grown in Pakistan. Pakistan Journal of Botany, 44(1), 231-236.
Khatoon	2007	2012	Maize	Aflatoxin B2	40	3	187	65	12	18.46	Asia	Pakistan		0.5	HPTLC	Khatoon, S., Hanif, N. Q., Tahira, I., Sultana, N., Sultana, K., & Ayub, N. (2012). Natural Occurrence of Aflatoxins, Zearalenone and Trichothecenes in Maize Grown in Pakistan. Pakistan Journal of Botany, 44(1), 231-236.
Khatoon	2007	2012	Maize	Aflatoxin G1	9	8	11	65	2	3	Asia	Pakistan		1	HPTLC	Khatoon, S., Hanif, N. Q., Tahira, I., Sultana, N., Sultana, K., & Ayub, N. (2012). Natural Occurrence of Aflatoxins, Zearalenone and Trichothecenes in Maize Grown in Pakistan. Pakistan Journal of Botany, 44(1), 231-236.
Khatoon	2007	2012	Maize	Zearalenone	1250	1250	1250	65	1	1.5	Asia	Pakistan		125	HPTLC	Khatoon, S., Hanif, N. Q., Tahira, I., Sultana, N., Sultana, K., & Ayub, N. (2012). Natural Occurrence of Aflatoxins, Zearalenone and Trichothecenes in Maize Grown in Pakistan. Pakistan Journal of Botany, 44(1), 231-236.
Khatoon	2007	2012	Maize	Deoxynivalenol	1549	136	2625	65	6	9.23	Asia	Pakistan		100	HPTLC	Khatoon, S., Hanif, N. Q., Tahira, I., Sultana, N., Sultana, K., & Ayub, N. (2012). Natural Occurrence of Aflatoxins, Zearalenone and Trichothecenes in Maize Grown in Pakistan. Pakistan Journal of Botany, 44(1), 231-236.
Khatoon	2007	2012	Maize	HT-2 toxin	236	100	500	65	4	6.15	Asia	Pakistan		100	HPTLC	Khatoon, S., Hanif, N. Q., Tahira, I., Sultana, N., Sultana, K., & Ayub, N. (2012). Natural Occurrence of Aflatoxins, Zearalenone and Trichothecenes in

																	Maize Grown in Pakistan. Pakistan Journal of Botany, 44(1), 231-236.
Khatoon	2007	2012	Maize	T-2 toxin			506	143	1125	65	4	6.15	Asia	Pakistan	100	HPTLC	Khatoon, S., Hanif, N. Q., Tahira, I., Sultana, N., Sultana, K., & Ayub, N. (2012). Natural Occurrence of Aflatoxins, Zearalenone and Trichothecenes in Maize Grown in Pakistan. Pakistan Journal of Botany, 44(1), 231-236.
Oruc	2010	2012	feed commodities	Aflatoxin B1	13.851	2.190	8.26	0.3	70	40	40	100	Asia	Turkey		ELISA	Oruc, H. H., Sorucu, A., Turkmen, II, & Arslan, E. (2012). Determination of Various Mycotoxin Concentrations in the Feedstuffs and Feed Produced by A Feed Manufacturer in Turkey. Kafkas Universitesi Veteriner Fakultesi Dergisi, 18(4), 633-638.
Oruc	2010	2012	feed commodities	T-2 toxin	11.827	1.870	30.4			40	40	100	Asia	Turkey		ELISA	Oruc, H. H., Sorucu, A., Turkmen, II, & Arslan, E. (2012). Determination of Various Mycotoxin Concentrations in the Feedstuffs and Feed Produced by A Feed Manufacturer in Turkey. Kafkas Universitesi Veteriner Fakultesi Dergisi, 18(4), 633-638.
Oruc	2010	2012	feed commodities	Fumonisins	1517.893	240.000	510			40	32	80	Asia	Turkey		ELISA	Oruc, H. H., Sorucu, A., Turkmen, II, & Arslan, E. (2012). Determination of Various Mycotoxin Concentrations in the Feedstuffs and Feed Produced by A Feed Manufacturer in Turkey. Kafkas Universitesi Veteriner Fakultesi Dergisi, 18(4), 633-638.
Oruc	2010	2012	feed commodities	Deoxynivalenol	1138.420	180.000	720			40	30	75	Asia	Turkey		ELISA	Oruc, H. H., Sorucu, A., Turkmen, II, & Arslan, E. (2012). Determination of Various Mycotoxin Concentrations in the Feedstuffs and Feed Produced by A Feed Manufacturer in Turkey. Kafkas Universitesi Veteriner Fakultesi Dergisi, 18(4), 633-638.
Oruc	2010	2012	feed commodities	Zearalenone	96.133	15.200	55.4			40	13	33	Asia	Turkey		ELISA	Oruc, H. H., Sorucu, A., Turkmen, II, & Arslan, E. (2012). Determination of Various Mycotoxin Concentrations in the Feedstuffs and Feed Produced by A Feed Manufacturer in Turkey. Kafkas Universitesi Veteriner Fakultesi Dergisi, 18(4), 633-638.
Rashedi	2010	2012	Maize	Zearalenone			150			8	2	25	Asia	Iran		ELISA	Rashedi, M., Sohrabi, H. R., Ashjaazadeh, M. A., Azizi, H., & Rahimi, E. (2012). Zearalenone contamination in barley, corn, silage and wheat bran. Toxicology and Industrial Health, 28(9), 779-782. doi:10.1177/0748233711422733
Rashedi	2010	2012	Barley	Zearalenone			136			20	4	20	Asia	Iran		ELISA	Rashedi, M., Sohrabi, H. R., Ashjaazadeh, M. A., Azizi, H., & Rahimi, E. (2012). Zearalenone contamination in barley, corn, silage and wheat bran. Toxicology and Industrial Health, 28(9), 779-782. doi:10.1177/0748233711422733
Rashedi	2010	2012	Silage	Zearalenone			140			12	2	16	Asia	Iran		ELISA	Rashedi, M., Sohrabi, H. R., Ashjaazadeh, M. A., Azizi, H., & Rahimi, E. (2012). Zearalenone contamination in barley, corn, silage and wheat

																		bran. Toxicology and Industrial Health, 28(9), 779-782. doi:10.1177/0748233711422733
Rashedi	2010	2012	Wheat bran	Zearalenone			0			14	0	0	Asia	Iran			ELISA	Rashedi, M., Sohrabi, H. R., Ashjaazadeh, M. A., Azizi, H., & Rahimi, E. (2012). Zearalenone contamination in barley, corn, silage and wheat bran. Toxicology and Industrial Health, 28(9), 779-782. doi:10.1177/0748233711422733
Rashid	2009-2010	2012	broiler feed	Aflatoxin B1	24.985	2.550	47.64	10	166	96	88	91	Asia	Pakistan	3		TLC	Rashid, N., Bajwa, M. A., Rafeeq, M., Khan, M. A., Ahmad, Z., Tariq, M. M., . . . Abbas, F. (2012). Prevalence of Aflatoxin B-1 in Finished Commercial Broiler Feed from West Central Pakistan. Journal of Animal and Plant Sciences-Japs, 22(1), 6-10.
Ahsan	2008-2009	2013	Whear grains	Aflatoxin B1				6	45	33	15	45	Asia	Pakistan	0.1	0.35	HPLC	Ahsan, S., Batti, I. A., Hussain, Z., Bukhari, S. A., Naqvi, S. A. R., Khan, Z. A., & Asi, M. R. (2013). HPLC Determination of Aflatoxins in Wheat Grains Collected from Central Areas of the Punjab, Pakistan. Asian Journal of Chemistry, 25(13), 7463-7466. doi:10.14233/ajchem.2013.15836
Ahsan	2008-2009	2013	Whear grains	Aflatoxin B2				5	20	33	10	30	Asia	Pakistan	0.2	0.6	HPLC	Ahsan, S., Batti, I. A., Hussain, Z., Bukhari, S. A., Naqvi, S. A. R., Khan, Z. A., & Asi, M. R. (2013). HPLC Determination of Aflatoxins in Wheat Grains Collected from Central Areas of the Punjab, Pakistan. Asian Journal of Chemistry, 25(13), 7463-7466. doi:10.14233/ajchem.2013.15836
Ahsan	2008-2009	2013	Whear grains	Aflatoxin G1				2	16	33	14	42	Asia	Pakistan	0.1	0.35	HPLC	Ahsan, S., Batti, I. A., Hussain, Z., Bukhari, S. A., Naqvi, S. A. R., Khan, Z. A., & Asi, M. R. (2013). HPLC Determination of Aflatoxins in Wheat Grains Collected from Central Areas of the Punjab, Pakistan. Asian Journal of Chemistry, 25(13), 7463-7466. doi:10.14233/ajchem.2013.15836
Ahsan	2008-2009	2013	Whear grains	Aflatoxin G2				0.8	6	33	3	8	Asia	Pakistan	0.2	0.6	HPLC	Ahsan, S., Batti, I. A., Hussain, Z., Bukhari, S. A., Naqvi, S. A. R., Khan, Z. A., & Asi, M. R. (2013). HPLC Determination of Aflatoxins in Wheat Grains Collected from Central Areas of the Punjab, Pakistan. Asian Journal of Chemistry, 25(13), 7463-7466. doi:10.14233/ajchem.2013.15836
Ahsan	2008-2009	2013	Whear grains	Total Aflatoxins				5	80	33	19	58	Asia	Pakistan			HPLC	Ahsan, S., Batti, I. A., Hussain, Z., Bukhari, S. A., Naqvi, S. A. R., Khan, Z. A., & Asi, M. R. (2013). HPLC Determination of Aflatoxins in Wheat Grains Collected from Central Areas of the Punjab, Pakistan. Asian Journal of Chemistry, 25(13), 7463-7466. doi:10.14233/ajchem.2013.15836
Ahsan	2008-2009	2013	Whear grains	Total Aflatoxins	5.240	1.353	52			15	12	80	Asia	Pakistan			HPLC	Ahsan, S., Batti, I. A., Hussain, Z., Bukhari, S. A., Naqvi, S. A. R., Khan, Z. A., & Asi, M. R. (2013). HPLC Determination of Aflatoxins in Wheat Grains Collected from Central Areas of the Punjab, Pakistan. Asian Journal of Chemistry, 25(13), 7463-7466. doi:10.14233/ajchem.2013.15837
Ahsan	2008-2009	2013	Whear grains	Total Aflatoxins	6.050	1.562	64			15	13	87	Asia	Pakistan			HPLC	Ahsan, S., Batti, I. A., Hussain, Z., Bukhari, S. A., Naqvi, S. A. R., Khan, Z. A., & Asi, M. R. (2013). HPLC Determination of Aflatoxins in Wheat Grains

Ahsan	2008-2009	2013	Whear grains	Total Aflatoxins	3.050	0.964	85			10	8	80	Asia	Pakistan		HPLC	Collected from Central Areas of the Punjab, Pakistan. Asian Journal of Chemistry, 25(13), 7463-7466. doi:10.14233/ajchem.2013.15838<br>Ahsan, S., Batti, I. A., Hussain, Z., Bukhari, S. A., Naqvi, S. A. R., Khan, Z. A., & Asi, M. R. (2013). HPLC Determination of Aflatoxins in Wheat Grains Collected from Central Areas of the Punjab, Pakistan. Asian Journal of Chemistry, 25(13), 7463-7466. doi:10.14233/ajchem.2013.15839
Kocasari	2007-2008	2013	Animal feed	Total Aflatoxins	1.260	0.094	10.72	3.82	116.83	180	108	60	Asia	Turkey		ELISA	Kocasari, F. S., Mor, F., Oguz, M. N., & Oguz, F. K. (2013). Occurrence of mycotoxins in feed samples in Burdur Province, Turkey. Environ Monit Assess, 185(6), 4943-4949. doi:10.1007/s10661-012-2915-3
Kocasari	2007-2008	2013	Animal feed	T-2 toxin	0.880	0.066	8.87	3.85	52.36	180	85	47.2	Asia	Turkey		ELISA	Kocasari, F. S., Mor, F., Oguz, M. N., & Oguz, F. K. (2013). Occurrence of mycotoxins in feed samples in Burdur Province, Turkey. Environ Monit Assess, 185(6), 4943-4949. doi:10.1007/s10661-012-2915-3
Kocasari	2007-2008	2013	Animal feed	Ochratoxin A	0.340	0.025	4.48	1.01	15.85	180	84	46.7	Asia	Turkey		ELISA	Kocasari, F. S., Mor, F., Oguz, M. N., & Oguz, F. K. (2013). Occurrence of mycotoxins in feed samples in Burdur Province, Turkey. Environ Monit Assess, 185(6), 4943-4949. doi:10.1007/s10661-012-2915-3
Kocasari	2007-2008	2013	Animal feed	Deoxynivalenol	7.030	0.524	59.76	18.5	500	180	87	48.3	Asia	Turkey		ELISA	Kocasari, F. S., Mor, F., Oguz, M. N., & Oguz, F. K. (2013). Occurrence of mycotoxins in feed samples in Burdur Province, Turkey. Environ Monit Assess, 185(6), 4943-4949. doi:10.1007/s10661-012-2915-3
Kocasari	2007-2008	2013	Animal feed	Zearalenone	0.850	0.063	7.79	2.1	29.3	180	57	31.7	Asia	Turkey		ELISA	Kocasari, F. S., Mor, F., Oguz, M. N., & Oguz, F. K. (2013). Occurrence of mycotoxins in feed samples in Burdur Province, Turkey. Environ Monit Assess, 185(6), 4943-4949. doi:10.1007/s10661-012-2915-3
Kocasari	2007-2008	2013	Animal feed	Fumonisins	147.000	10.957	3190	2690	4960	180	19	10.6	Asia	Turkey		ELISA	Kocasari, F. S., Mor, F., Oguz, M. N., & Oguz, F. K. (2013). Occurrence of mycotoxins in feed samples in Burdur Province, Turkey. Environ Monit Assess, 185(6), 4943-4949. doi:10.1007/s10661-012-2915-3
Mahmoudi	2010-2011	2013	Maize	Total Aflatoxins	1.249	0.170	2.79	0.3	4.93	54	8	14.81	Asia	Iran	0.32	HPLC	Mahmoudi, R., Norian, R., Katiraee, F., & Pajohi Alamoti, M. R. (2013). Total aflatoxin contamination of maize produced in different regions of Qazvin-Iran. International Food Research Journal, 20(5), 2901-2904. Retrieved from https://www.scopus.com/inward/record.uri?eid=2-s2.0-84887508522&partnerID=40&md5=56bb79dc13dba8a25128eb9412793f7c
Sadegh		2013	Animal feed	Aflatoxin B1	2.140	0.216	2.311	0.34	5.81	98	33	36.67	Asia	Iran		HPLC	Sadegh, M., Sani, A. M., & Ghiasvand, R. (2013). Determination of aflatoxin B1 in animal feed in Mashhad, Iran. BioTechnology: An Indian Journal, 7(9), 334-336. Retrieved from https://www.scopus.com/inward/record.uri?eid=2-s2.0-84887166842&partnerID=40&md5=c94e3c7eab055e8304c50d6594580ded

Bilal	2011	2014	feed commodities	Aflatoxin B1		1.02	0	11.37	76	20	26.32	Asia	Turkey		0.2	HPLC	Bilal, T., Aksakal, D. H., Sunnetci, S., Keser, O., & Eseceli, H. (2014). Detection of Aflatoxin, Zearalenone and Deoxynivalenol in Some Feed and Feedstuffs in Turkey. Pakistan Veterinary Journal, 34(4), 459-463.
Bilal	2011	2014	feed commodities	Aflatoxin B2		0.13	0	1.76	76	6	7.89	Asia	Turkey		0.1	HPLC	Bilal, T., Aksakal, D. H., Sunnetci, S., Keser, O., & Eseceli, H. (2014). Detection of Aflatoxin, Zearalenone and Deoxynivalenol in Some Feed and Feedstuffs in Turkey. Pakistan Veterinary Journal, 34(4), 459-463.
Bilal	2011	2014	feed commodities	Aflatoxin G1		0.04	0	1.9	76	4	5.26	Asia	Turkey		0.3	HPLC	Bilal, T., Aksakal, D. H., Sunnetci, S., Keser, O., & Eseceli, H. (2014). Detection of Aflatoxin, Zearalenone and Deoxynivalenol in Some Feed and Feedstuffs in Turkey. Pakistan Veterinary Journal, 34(4), 459-463.
Bilal	2011	2014	feed commodities	Aflatoxin G2		0.01	0	0.04	76	4	5.26	Asia	Turkey		0.5	HPLC	Bilal, T., Aksakal, D. H., Sunnetci, S., Keser, O., & Eseceli, H. (2014). Detection of Aflatoxin, Zearalenone and Deoxynivalenol in Some Feed and Feedstuffs in Turkey. Pakistan Veterinary Journal, 34(4), 459-463.
Bilal	2011	2014	Animal feed	Aflatoxin B1		0.26	0	3.31	30	17	56.6	Asia	Turkey		0.6	HPLC	Bilal, T., Aksakal, D. H., Sunnetci, S., Keser, O., & Eseceli, H. (2014). Detection of Aflatoxin, Zearalenone and Deoxynivalenol in Some Feed and Feedstuffs in Turkey. Pakistan Veterinary Journal, 34(4), 459-463.
Bilal	2011	2014	Animal feed	Aflatoxin B2		0.01	0	0.31	30	1	3.33	Asia	Turkey		0.4	HPLC	Bilal, T., Aksakal, D. H., Sunnetci, S., Keser, O., & Eseceli, H. (2014). Detection of Aflatoxin, Zearalenone and Deoxynivalenol in Some Feed and Feedstuffs in Turkey. Pakistan Veterinary Journal, 34(4), 459-463.
Bilal	2011	2014	Animal feed	Aflatoxin G1		0.3	0	1.1	30	9	30	Asia	Turkey		0.8	HPLC	Bilal, T., Aksakal, D. H., Sunnetci, S., Keser, O., & Eseceli, H. (2014). Detection of Aflatoxin, Zearalenone and Deoxynivalenol in Some Feed and Feedstuffs in Turkey. Pakistan Veterinary Journal, 34(4), 459-463.
Bilal	2011	2014	Animal feed	Aflatoxin G2		0.11	0	0.67	30	1	3.3	Asia	Turkey		0.8	HPLC	Bilal, T., Aksakal, D. H., Sunnetci, S., Keser, O., & Eseceli, H. (2014). Detection of Aflatoxin, Zearalenone and Deoxynivalenol in Some Feed and Feedstuffs in Turkey. Pakistan Veterinary Journal, 34(4), 459-463.
Bilal	2011	2014	feed commodities	Zearalenone		7.34	0	96.61	76	24	31.58	Asia	Turkey		15	HPLC	Bilal, T., Aksakal, D. H., Sunnetci, S., Keser, O., & Eseceli, H. (2014). Detection of Aflatoxin, Zearalenone and Deoxynivalenol in Some Feed and Feedstuffs in Turkey. Pakistan Veterinary Journal, 34(4), 459-463.
Bilal	2011	2014	feed commodities	Deoxynivalenol		423.17	0	4769.63	76	14	18.42	Asia	Turkey		250	HPLC	Bilal, T., Aksakal, D. H., Sunnetci, S., Keser, O., & Eseceli, H. (2014). Detection of Aflatoxin, Zearalenone and Deoxynivalenol in Some Feed and Feedstuffs in Turkey. Pakistan Veterinary Journal, 34(4), 459-463.

Bilal	2011	2014	Animal feed	Zearalenone			13.4	0	37.72	30	22	73.33	Asia	Turkey		15	HPLC	Bilal, T., Aksakal, D. H., Sunnetci, S., Keser, O., & Eseceli, H. (2014). Detection of Aflatoxin, Zearalenone and Deoxynivalenol in Some Feed and Feedstuffs in Turkey. Pakistan Veterinary Journal, 34(4), 459-463.
Bilal	2011	2014	Animal feed	Deoxynivalenol			62.85	0	486.2	30	13	43.33	Asia	Turkey		200	HPLC	Bilal, T., Aksakal, D. H., Sunnetci, S., Keser, O., & Eseceli, H. (2014). Detection of Aflatoxin, Zearalenone and Deoxynivalenol in Some Feed and Feedstuffs in Turkey. Pakistan Veterinary Journal, 34(4), 459-463.
Eskandari	2012	2014	Animal feed	Aflatoxin B1			26.4	15	35	40	40	100	Asia	Iran	0.05		HPLC	Eskandari, M. H., & Pakfetrat, S. (2014). Aflatoxins and heavy metals in animal feed in Iran. Food Addit Contam Part B Surveill, 7(3), 202-207. doi:10.1080/19393210.2013.876675
Eskandari	2012	2014	Animal feed	Aflatoxin B2			15.4	5	25	40	40	100	Asia	Iran	0.015		HPLC	Eskandari, M. H., & Pakfetrat, S. (2014). Aflatoxins and heavy metals in animal feed in Iran. Food Addit Contam Part B Surveill, 7(3), 202-207. doi:10.1080/19393210.2013.876675
Eskandari	2012	2014	Animal feed	Aflatoxin G1			11.7		30	40	38	95	Asia	Iran	0.03		HPLC	Eskandari, M. H., & Pakfetrat, S. (2014). Aflatoxins and heavy metals in animal feed in Iran. Food Addit Contam Part B Surveill, 7(3), 202-207. doi:10.1080/19393210.2013.876675
Eskandari	2012	2014	Animal feed	Aflatoxin G2			4.9		16	40	30	75	Asia	Iran	0.015		HPLC	Eskandari, M. H., & Pakfetrat, S. (2014). Aflatoxins and heavy metals in animal feed in Iran. Food Addit Contam Part B Surveill, 7(3), 202-207. doi:10.1080/19393210.2013.876675
Eskandari	2012	2014	Animal feed	Total Aflatoxins			58.5	28	90	40	40	100	Asia	Iran			HPLC	Eskandari, M. H., & Pakfetrat, S. (2014). Aflatoxins and heavy metals in animal feed in Iran. Food Addit Contam Part B Surveill, 7(3), 202-207. doi:10.1080/19393210.2013.876675
Gholampour Azizi	2013	2014	Cattle feed commodities	T-2 toxin	11.690	1.509	11.45	0.1	53.5	60	7	11.7	Asia	Iran			ELISA	Gholampour Azizi, I., Azarmi, M., Danesh Pouya, N., & Rouhi, S. (2014). T-2 toxin Analysis in Poultry and Cattle Feedstuff. Jundishapur J Nat Pharm Prod, 9(2), e13734. doi:10.17795/jjnpp-13734
Gholampour Azizi	2013	2014	poultry feed commodities	T-2 toxin	10.160	1.855	11.76	0.1	27.5	30	3	10	Asia	Iran			ELISA	Gholampour Azizi, I., Azarmi, M., Danesh Pouya, N., & Rouhi, S. (2014). T-2 toxin Analysis in Poultry and Cattle Feedstuff. Jundishapur J Nat Pharm Prod, 9(2), e13734. doi:10.17795/jjnpp-13734
Shar		2014	Layer feed	Total Aflatoxins			20		72	43	22	51	Asia	Pakistan	0.5	2	HPLC	Shar, Z. H., Sumbal, G. A., Sherazi, S. T., Bhanger, M. I., & Nizamani, S. M. (2014). Natural co-occurrence of aflatoxins and deoxynivalenol in poultry feed in Pakistan. Food Addit Contam Part B Surveill, 7(3), 162-167. doi:10.1080/19393210.2013.867904
Shar		2014	Broiler Starter (Mesh)	Total Aflatoxins			19		55	38	15	39	Asia	Pakistan	0.5	2	HPLC	Shar, Z. H., Sumbal, G. A., Sherazi, S. T., Bhanger, M. I., & Nizamani, S. M. (2014). Natural co-occurrence of aflatoxins and deoxynivalenol in poultry feed in Pakistan. Food Addit Contam Part B Surveill, 7(3), 162-167. doi:10.1080/19393210.2013.867904

Shar		2014	Broiler Finisher (Mesh)	Total Aflatoxins			20		50	39	17	43	Asia	Pakistan	0.5	2	HPLC	Shar, Z. H., Sumbal, G. A., Sherazi, S. T., Bhanger, M. I., & Nizamani, S. M. (2014). Natural co-occurrence of aflatoxins and deoxynivalenol in poultry feed in Pakistan. Food Addit Contam Part B Surveill, 7(3), 162-167. doi:10.1080/19393210.2013.867904
Shar		2014	Broiler Starter (Crumb)	Total Aflatoxins			27		77	47	25	53	Asia	Pakistan	0.5	2	HPLC	Shar, Z. H., Sumbal, G. A., Sherazi, S. T., Bhanger, M. I., & Nizamani, S. M. (2014). Natural co-occurrence of aflatoxins and deoxynivalenol in poultry feed in Pakistan. Food Addit Contam Part B Surveill, 7(3), 162-167. doi:10.1080/19393210.2013.867904
Shar		2014	Broiler Finisher (Crumb)	Total Aflatoxins			25		80	48	21	43	Asia	Pakistan	0.5	2	HPLC	Shar, Z. H., Sumbal, G. A., Sherazi, S. T., Bhanger, M. I., & Nizamani, S. M. (2014). Natural co-occurrence of aflatoxins and deoxynivalenol in poultry feed in Pakistan. Food Addit Contam Part B Surveill, 7(3), 162-167. doi:10.1080/19393210.2013.867904
Shar		2014	Layer feed	Deoxynivalenol			671		1200	43	24	48	Asia	Pakistan	5	15	HPLC	Shar, Z. H., Sumbal, G. A., Sherazi, S. T., Bhanger, M. I., & Nizamani, S. M. (2014). Natural co-occurrence of aflatoxins and deoxynivalenol in poultry feed in Pakistan. Food Addit Contam Part B Surveill, 7(3), 162-167. doi:10.1080/19393210.2013.867904
Shar		2014	Broiler Starter (Mesh)	Deoxynivalenol			592		900	38	17	44	Asia	Pakistan	5	15	HPLC	Shar, Z. H., Sumbal, G. A., Sherazi, S. T., Bhanger, M. I., & Nizamani, S. M. (2014). Natural co-occurrence of aflatoxins and deoxynivalenol in poultry feed in Pakistan. Food Addit Contam Part B Surveill, 7(3), 162-167. doi:10.1080/19393210.2013.867904
Shar		2014	Broiler Finisher (Mesh)	Deoxynivalenol			573		1250	39	21	53	Asia	Pakistan	5	15	HPLC	Shar, Z. H., Sumbal, G. A., Sherazi, S. T., Bhanger, M. I., & Nizamani, S. M. (2014). Natural co-occurrence of aflatoxins and deoxynivalenol in poultry feed in Pakistan. Food Addit Contam Part B Surveill, 7(3), 162-167. doi:10.1080/19393210.2013.867904
Shar		2014	Broiler Starter (Crumb)	Deoxynivalenol			798		1400	47	30	63	Asia	Pakistan	5	15	HPLC	Shar, Z. H., Sumbal, G. A., Sherazi, S. T., Bhanger, M. I., & Nizamani, S. M. (2014). Natural co-occurrence of aflatoxins and deoxynivalenol in poultry feed in Pakistan. Food Addit Contam Part B Surveill, 7(3), 162-167. doi:10.1080/19393210.2013.867904
Shar		2014	Broiler Finisher (Crumb)	Deoxynivalenol			940		1600	48	22	45	Asia	Pakistan	5	15	HPLC	Shar, Z. H., Sumbal, G. A., Sherazi, S. T., Bhanger, M. I., & Nizamani, S. M. (2014). Natural co-occurrence of aflatoxins and deoxynivalenol in poultry feed in Pakistan. Food Addit Contam Part B Surveill, 7(3), 162-167. doi:10.1080/19393210.2013.867904
Sherazi	2013-2014	2015	Wheat	Ochratoxin A	44.700	7.556	65.1	10	134	35	9	36	Asia	Pakistan	0.2	0.6	HPLC	Sherazi, S. T., Shar, Z. H., Sumbal, G. A., Tan, E. T., Bhanger, M. I., Kara, H., & Nizamani, S. M. (2015). Occurrence of ochratoxin A in poultry feeds and

																	feed ingredients from Pakistan. Mycotoxin Res, 31(1), 1-7. doi:10.1007/s12550-014-0216-0
Sherazi	2013-2014	2015	Maize	Ochratoxin A	58.700	10.717	80.8	18	180	30	12	40	Asia	Pakistan	0.2	0.6	HPLC	Sherazi, S. T., Shar, Z. H., Sumbal, G. A., Tan, E. T., Bhanger, M. I., Kara, H., & Nizamani, S. M. (2015). Occurrence of ochratoxin A in poultry feeds and feed ingredients from Pakistan. Mycotoxin Res, 31(1), 1-7. doi:10.1007/s12550-014-0216-0
Sherazi	2013-2014	2015	Rice	Ochratoxin A	35.400	6.463	57.2	10	120	30	9	30	Asia	Pakistan	0.2	0.6	HPLC	Sherazi, S. T., Shar, Z. H., Sumbal, G. A., Tan, E. T., Bhanger, M. I., Kara, H., & Nizamani, S. M. (2015). Occurrence of ochratoxin A in poultry feeds and feed ingredients from Pakistan. Mycotoxin Res, 31(1), 1-7. doi:10.1007/s12550-014-0216-0
Sherazi	2013-2014	2015	Sorghum	Ochratoxin A	48.800	10.404	50	17	115	22	7	31.8	Asia	Pakistan	0.2	0.6	HPLC	Sherazi, S. T., Shar, Z. H., Sumbal, G. A., Tan, E. T., Bhanger, M. I., Kara, H., & Nizamani, S. M. (2015). Occurrence of ochratoxin A in poultry feeds and feed ingredients from Pakistan. Mycotoxin Res, 31(1), 1-7. doi:10.1007/s12550-014-0216-0
Sherazi	2013-2014	2015	Sunflower meal	Ochratoxin A	37.900	8.270	52.5	14	60	21	6	28.5	Asia	Pakistan	0.2	0.6	HPLC	Sherazi, S. T., Shar, Z. H., Sumbal, G. A., Tan, E. T., Bhanger, M. I., Kara, H., & Nizamani, S. M. (2015). Occurrence of ochratoxin A in poultry feeds and feed ingredients from Pakistan. Mycotoxin Res, 31(1), 1-7. doi:10.1007/s12550-014-0216-0
Sherazi	2013-2014	2015	Guar meal	Ochratoxin A	19.200	4.657	39.3	15	60	17	6	35.2	Asia	Pakistan	0.2	0.6	HPLC	Sherazi, S. T., Shar, Z. H., Sumbal, G. A., Tan, E. T., Bhanger, M. I., Kara, H., & Nizamani, S. M. (2015). Occurrence of ochratoxin A in poultry feeds and feed ingredients from Pakistan. Mycotoxin Res, 31(1), 1-7. doi:10.1007/s12550-014-0216-0
Sherazi	2013-2014	2015	Cotton Seed meal	Ochratoxin A	21.900	5.162	41	19	80	18	5	27.7	Asia	Pakistan	0.2	0.6	HPLC	Sherazi, S. T., Shar, Z. H., Sumbal, G. A., Tan, E. T., Bhanger, M. I., Kara, H., & Nizamani, S. M. (2015). Occurrence of ochratoxin A in poultry feeds and feed ingredients from Pakistan. Mycotoxin Res, 31(1), 1-7. doi:10.1007/s12550-014-0216-0
Sherazi	2013-2014	2015	Corn gluten meal	Ochratoxin A	24.100	5.529	53.8	12	35	19	6	31.5	Asia	Pakistan	0.2	0.6	HPLC	Sherazi, S. T., Shar, Z. H., Sumbal, G. A., Tan, E. T., Bhanger, M. I., Kara, H., & Nizamani, S. M. (2015). Occurrence of ochratoxin A in poultry feeds and feed ingredients from Pakistan. Mycotoxin Res, 31(1), 1-7. doi:10.1007/s12550-014-0216-0
Sherazi	2013-2014	2015	Wheat bran	Ochratoxin A	10.100	2.153	22.5	12	35	22	6	27.2	Asia	Pakistan	0.2	0.6	HPLC	Sherazi, S. T., Shar, Z. H., Sumbal, G. A., Tan, E. T., Bhanger, M. I., Kara, H., & Nizamani, S. M. (2015). Occurrence of ochratoxin A in poultry feeds and feed ingredients from Pakistan. Mycotoxin Res, 31(1), 1-7. doi:10.1007/s12550-014-0216-0
Sherazi	2013-2014	2015	Barley	Ochratoxin A	54.600	9.969	65.2	20	160	30	10	33.3	Asia	Pakistan	0.2	0.6	HPLC	Sherazi, S. T., Shar, Z. H., Sumbal, G. A., Tan, E. T., Bhanger, M. I., Kara, H., & Nizamani, S. M. (2015). Occurrence of ochratoxin A in poultry feeds and feed ingredients from Pakistan. Mycotoxin Res, 31(1), 1-7. doi:10.1007/s12550-014-0216-0
Sherazi	2013-2014	2015	Peanut meal	Ochratoxin A	33.700	8.425	53.8	14	110	16	5	31.2	Asia	Pakistan	0.2	0.6	HPLC	Sherazi, S. T., Shar, Z. H., Sumbal, G. A., Tan, E. T., Bhanger, M. I., Kara, H., & Nizamani, S. M. (2015). Occurrence of ochratoxin A in poultry feeds and

																		feed ingredients from Pakistan. Mycotoxin Res, 31(1), 1-7. doi:10.1007/s12550-014-0216-0
Sherazi	2013-2014	2015	Millet	Ochratoxin A	23.600	5.563	37.8	15	65	18	4	22.2	Asia	Pakistan	0.2	0.6	HPLC	Sherazi, S. T., Shar, Z. H., Sumbal, G. A., Tan, E. T., Bhanger, M. I., Kara, H., & Nizamani, S. M. (2015). Occurrence of ochratoxin A in poultry feeds and feed ingredients from Pakistan. Mycotoxin Res, 31(1), 1-7. doi:10.1007/s12550-014-0216-0
Sherazi	2013-2014	2015	Broiler Starter (Mesh)	Ochratoxin A	49.300	11.024	66	22	150	20	6	30	Asia	Pakistan	0.2	0.6	HPLC	Sherazi, S. T., Shar, Z. H., Sumbal, G. A., Tan, E. T., Bhanger, M. I., Kara, H., & Nizamani, S. M. (2015). Occurrence of ochratoxin A in poultry feeds and feed ingredients from Pakistan. Mycotoxin Res, 31(1), 1-7. doi:10.1007/s12550-014-0216-0
Sherazi	2013-2014	2015	Broiler Finisher (Mesh)	Ochratoxin A	44.300	9.906	56	22	157	20	9	45	Asia	Pakistan	0.2	0.6	HPLC	Sherazi, S. T., Shar, Z. H., Sumbal, G. A., Tan, E. T., Bhanger, M. I., Kara, H., & Nizamani, S. M. (2015). Occurrence of ochratoxin A in poultry feeds and feed ingredients from Pakistan. Mycotoxin Res, 31(1), 1-7. doi:10.1007/s12550-014-0216-0
Sherazi	2013-2014	2015	Broiler Starter (Crumb)	Ochratoxin A	41.200	9.213	79	30	140	20	8	40	Asia	Pakistan	0.2	0.6	HPLC	Sherazi, S. T., Shar, Z. H., Sumbal, G. A., Tan, E. T., Bhanger, M. I., Kara, H., & Nizamani, S. M. (2015). Occurrence of ochratoxin A in poultry feeds and feed ingredients from Pakistan. Mycotoxin Res, 31(1), 1-7. doi:10.1007/s12550-014-0216-0
Sherazi	2013-2014	2015	Broiler Finisher (Crumb)	Ochratoxin A	67.000	14.982	85	22	190	20	7	35	Asia	Pakistan	0.2	0.6	HPLC	Sherazi, S. T., Shar, Z. H., Sumbal, G. A., Tan, E. T., Bhanger, M. I., Kara, H., & Nizamani, S. M. (2015). Occurrence of ochratoxin A in poultry feeds and feed ingredients from Pakistan. Mycotoxin Res, 31(1), 1-7. doi:10.1007/s12550-014-0216-0
Namjoo	2009-2010	2016	Wheat Silage	Total Aflatoxins	0.670	0.115	1.83		7.08	34	10	29	Asia	Iran			HPLC	NAMJOO, M., SALAMAT, F., RAJABLI, N., HAJIHOSEEINI, R., NIKNEJAD, F., KOHSAR, F. & JOSHAGHANI, H. 2016. Quantitative Determination of Aflatoxin by High Performance Liquid Chromatography in Wheat Silos in Golestan Province, North of Iran. Iran J Public Health, 45, 905-10.
Asghar	2014	2016	Wheat	Aflatoxin B1	0.320	0.024	0.51	0.05	4.78	185	48	26	Asia	Pakistan	0.031	0.093	HPLC	Asghar, M. A., Ahmed, A., Iqbal, J., Zahir, E., & Nauman, H. (2016). Fungal flora and aflatoxin contamination in Pakistani wheat kernels (Triticum aestivum L.) and their attribution in seed germination. J Food Drug Anal, 24(3), 635-643. doi:10.1016/j.jfda.2016.02.001
Asghar	2014	2016	Wheat	Aflatoxin B2	0.080	0.006	0.02	0.02	0.48	185	13	7	Asia	Pakistan	0.022	0.066	HPLC	Asghar, M. A., Ahmed, A., Iqbal, J., Zahir, E., & Nauman, H. (2016). Fungal flora and aflatoxin contamination in Pakistani wheat kernels (Triticum aestivum L.) and their attribution in seed germination. J Food Drug Anal, 24(3), 635-643. doi:10.1016/j.jfda.2016.02.001
Asghar	2014	2016	Wheat	Aflatoxin G1			0	0	0	185	0	0	Asia	Pakistan	0.032	0.096	HPLC	Asghar, M. A., Ahmed, A., Iqbal, J., Zahir, E., & Nauman, H. (2016). Fungal flora and aflatoxin contamination in Pakistani wheat kernels (Triticum aestivum L.) and their attribution in seed

																		germination. J Food Drug Anal, 24(3), 635-643. doi:10.1016/j.jfda.2016.02.002
Asghar	2014	2016	Wheat	Aflatoxin G2			0	0	0	185	0	0	Asia	Pakistan	0.028	0.084	HPLC	Asghar, M. A., Ahmed, A., Iqbal, J., Zahir, E., & Nauman, H. (2016). Fungal flora and aflatoxin contamination in Pakistani wheat kernels (Triticum aestivum L.) and their attribution in seed germination. J Food Drug Anal, 24(3), 635-643. doi:10.1016/j.jfda.2016.02.003
Asghar	2014	2016	Wheat	Total Aflatoxins	0.400	0.029	0.53	0.02	5.26	185	48	26	Asia	Pakistan	0.091	0.273	HPLC	Asghar, M. A., Ahmed, A., Iqbal, J., Zahir, E., & Nauman, H. (2016). Fungal flora and aflatoxin contamination in Pakistani wheat kernels (Triticum aestivum L.) and their attribution in seed germination. J Food Drug Anal, 24(3), 635-643. doi:10.1016/j.jfda.2016.02.001
Bahrami	2014	2016	Barley	Aflatoxin B1	0.270	0.043	0.6	0.12	1.01	40	36	90	Asia	Iran	0.12	0.4	HPLC	Bahrami, R., Shahbazi, Y., & Nikousefat, Z. (2016). Occurrence and seasonal variation of aflatoxin in dairy cow feed with estimation of aflatoxin M-1 in milk from Iran. Food and Agricultural Immunology, 27(3), 388-400. doi:10.1080/09540105.2015.1109613
Bahrami	2014	2016	Barley	Aflatoxin B2	0.190	0.030	0.24	0.015	0.61	40	29	72.5	Asia	Iran	0.015	0.05	HPLC	Bahrami, R., Shahbazi, Y., & Nikousefat, Z. (2016). Occurrence and seasonal variation of aflatoxin in dairy cow feed with estimation of aflatoxin M-1 in milk from Iran. Food and Agricultural Immunology, 27(3), 388-400. doi:10.1080/09540105.2015.1109613
Bahrami	2014	2016	Barley	Aflatoxin G1	0.100	0.016	0.13	0.05	0.41	40	19	47.5	Asia	Iran	0.05	0.16	HPLC	Bahrami, R., Shahbazi, Y., & Nikousefat, Z. (2016). Occurrence and seasonal variation of aflatoxin in dairy cow feed with estimation of aflatoxin M-1 in milk from Iran. Food and Agricultural Immunology, 27(3), 388-400. doi:10.1080/09540105.2015.1109613
Bahrami	2014	2016	Barley	Aflatoxin G2	0.050	0.008	0.07	0.03	0.2	40	19	47.5	Asia	Iran	0.03	0.1	HPLC	Bahrami, R., Shahbazi, Y., & Nikousefat, Z. (2016). Occurrence and seasonal variation of aflatoxin in dairy cow feed with estimation of aflatoxin M-1 in milk from Iran. Food and Agricultural Immunology, 27(3), 388-400. doi:10.1080/09540105.2015.1109613
Bahrami	2014	2016	Corn silage	Aflatoxin G1	1.870	0.296	2.6	0.05	6.04	40	28	70	Asia	Iran	0.05	0.16	HPLC	Bahrami, R., Shahbazi, Y., & Nikousefat, Z. (2016). Occurrence and seasonal variation of aflatoxin in dairy cow feed with estimation of aflatoxin M-1 in milk from Iran. Food and Agricultural Immunology, 27(3), 388-400. doi:10.1080/09540105.2015.1109614
Bahrami	2014	2016	Corn silage	Aflatoxin G2	0.930	0.147	1.3	0.03	2.9	40	28	70	Asia	Iran	0.03	0.1	HPLC	Bahrami, R., Shahbazi, Y., & Nikousefat, Z. (2016). Occurrence and seasonal variation of aflatoxin in dairy cow feed with estimation of aflatoxin M-1 in milk from Iran. Food and Agricultural Immunology, 27(3), 388-400. doi:10.1080/09540105.2015.1109615

Bahrami	2014	2016	Corn silage	Aflatoxin B1	2.370	0.375	4.47	0.3	8.24	40	40	100	Asia	Iran	0.12	0.4	HPLC	Bahrami, R., Shahbazi, Y., & Nikousefat, Z. (2016). Occurrence and seasonal variation of aflatoxin in dairy cow feed with estimation of aflatoxin M-1 in milk from Iran. Food and Agricultural Immunology, 27(3), 388-400. doi:10.1080/09540105.2015.1109616
Bahrami	2014	2016	Corn silage	Aflatoxin B2	2.270	0.359	3.53	0.015	7.24	40	32	80	Asia	Iran	0.015	0.05	HPLC	Bahrami, R., Shahbazi, Y., & Nikousefat, Z. (2016). Occurrence and seasonal variation of aflatoxin in dairy cow feed with estimation of aflatoxin M-1 in milk from Iran. Food and Agricultural Immunology, 27(3), 388-400. doi:10.1080/09540105.2015.1109617
Bahrami	2014	2016	Alfalfa hay	Aflatoxin G1	0.030	0.005	0.06	0.05	0.18	40	10	25	Asia	Iran	0.05	0.16	HPLC	Bahrami, R., Shahbazi, Y., & Nikousefat, Z. (2016). Occurrence and seasonal variation of aflatoxin in dairy cow feed with estimation of aflatoxin M-1 in milk from Iran. Food and Agricultural Immunology, 27(3), 388-400. doi:10.1080/09540105.2015.1109618
Bahrami	2014	2016	Alfalfa hay	Aflatoxin G2	0.140	0.022	0.03	0.03	0.09	40	8	20	Asia	Iran	0.03	0.1	HPLC	Bahrami, R., Shahbazi, Y., & Nikousefat, Z. (2016). Occurrence and seasonal variation of aflatoxin in dairy cow feed with estimation of aflatoxin M-1 in milk from Iran. Food and Agricultural Immunology, 27(3), 388-400. doi:10.1080/09540105.2015.1109619
Bahrami	2014	2016	Alfalfa hay	Aflatoxin B1	0.150	0.024	0.2	0.12	0.48	40	24	60	Asia	Iran	0.12	0.4	HPLC	Bahrami, R., Shahbazi, Y., & Nikousefat, Z. (2016). Occurrence and seasonal variation of aflatoxin in dairy cow feed with estimation of aflatoxin M-1 in milk from Iran. Food and Agricultural Immunology, 27(3), 388-400. doi:10.1080/09540105.2015.1109620
Bahrami	2014	2016	Alfalfa hay	Aflatoxin B2	0.120	0.019	0.13	0.015	0.38	40	24	60	Asia	Iran	0.015	0.05	HPLC	Bahrami, R., Shahbazi, Y., & Nikousefat, Z. (2016). Occurrence and seasonal variation of aflatoxin in dairy cow feed with estimation of aflatoxin M-1 in milk from Iran. Food and Agricultural Immunology, 27(3), 388-400. doi:10.1080/09540105.2015.1109621
Bahrami	2014	2016	Straw	Aflatoxin G1	0.970	0.153	0.5	0.05	3.5	40	12	30	Asia	Iran	0.05	0.16	HPLC	Bahrami, R., Shahbazi, Y., & Nikousefat, Z. (2016). Occurrence and seasonal variation of aflatoxin in dairy cow feed with estimation of aflatoxin M-1 in milk from Iran. Food and Agricultural Immunology, 27(3), 388-400. doi:10.1080/09540105.2015.1109622
Bahrami	2014	2016	Straw	Aflatoxin G2	0.480	0.076	0.26	0.03	1.75	40	12	30	Asia	Iran	0.03	0.1	HPLC	Bahrami, R., Shahbazi, Y., & Nikousefat, Z. (2016). Occurrence and seasonal variation of aflatoxin in dairy cow feed with estimation of aflatoxin M-1 in milk from Iran. Food and Agricultural Immunology, 27(3), 388-400. doi:10.1080/09540105.2015.1109623
Bahrami	2014	2016	Straw	Aflatoxin B1	1.500	0.237	1.72	0.12	5.5	40	32	80	Asia	Iran	0.12	0.4	HPLC	Bahrami, R., Shahbazi, Y., & Nikousefat, Z. (2016). Occurrence and seasonal variation of aflatoxin in dairy cow feed with estimation of aflatoxin M-1 in

																		milk from Iran. Food and Agricultural Immunology, 27(3), 388-400. doi:10.1080/09540105.2015.1109624
Bahrami	2014	2016	Straw	Aflatoxin B2	1.310	0.207	0.95	0.015	4.5	40	26	65	Asia	Iran	0.015	0.05	HPLC	Bahrami, R., Shahbazi, Y., & Nikousefat, Z. (2016). Occurrence and seasonal variation of aflatoxin in dairy cow feed with estimation of aflatoxin M-1 in milk from Iran. Food and Agricultural Immunology, 27(3), 388-400. doi:10.1080/09540105.2015.1109625
Chohan	2013-2014	2016	Maize	Aflatoxin B1	10.462	1.720	28.18	2.56	59.96	37	12	32	Asia	Pakistan			ELISA	Chohan, K. A., Awan, F., Ali, M. M., Iqbal, U., & Ijaz, M. (2016). Assessment of Aflatoxin in Dairy Concentrate Feeds, Total Mixed Rations, Silage and Various Feed Ingredients in Pakistan. Pakistan Journal of Zoology, 48(1), 277-280.
Chohan	2013-2014	2016	Beet pulp	Aflatoxin B1	0.280	0.140	15.89	9.52	25.01	4	1	25	Asia	Pakistan			ELISA	Chohan, K. A., Awan, F., Ali, M. M., Iqbal, U., & Ijaz, M. (2016). Assessment of Aflatoxin in Dairy Concentrate Feeds, Total Mixed Rations, Silage and Various Feed Ingredients in Pakistan. Pakistan Journal of Zoology, 48(1), 277-280.
Chohan	2013-2014	2016	Cotton seed cake	Aflatoxin B1	46.800	9.360	111.94	6.92	185.97	25	17	68	Asia	Pakistan			ELISA	Chohan, K. A., Awan, F., Ali, M. M., Iqbal, U., & Ijaz, M. (2016). Assessment of Aflatoxin in Dairy Concentrate Feeds, Total Mixed Rations, Silage and Various Feed Ingredients in Pakistan. Pakistan Journal of Zoology, 48(1), 277-280.
Chohan	2013-2014	2016	Soya bean meal	Aflatoxin B1	6.409	1.850	19.2	3.56	38.45	12	2	16	Asia	Pakistan			ELISA	Chohan, K. A., Awan, F., Ali, M. M., Iqbal, U., & Ijaz, M. (2016). Assessment of Aflatoxin in Dairy Concentrate Feeds, Total Mixed Rations, Silage and Various Feed Ingredients in Pakistan. Pakistan Journal of Zoology, 48(1), 277-280.
Chohan	2013-2014	2016	Rice	Aflatoxin B1	6.513	1.880	16.4	1.02	30.98	12	6	50	Asia	Pakistan			ELISA	Chohan, K. A., Awan, F., Ali, M. M., Iqbal, U., & Ijaz, M. (2016). Assessment of Aflatoxin in Dairy Concentrate Feeds, Total Mixed Rations, Silage and Various Feed Ingredients in Pakistan. Pakistan Journal of Zoology, 48(1), 277-280.
Chohan	2013-2014	2016	Corn gluten meal	Aflatoxin B1	55.498	17.550	116.77	17.14	210.07	10	6	60	Asia	Pakistan			ELISA	Chohan, K. A., Awan, F., Ali, M. M., Iqbal, U., & Ijaz, M. (2016). Assessment of Aflatoxin in Dairy Concentrate Feeds, Total Mixed Rations, Silage and Various Feed Ingredients in Pakistan. Pakistan Journal of Zoology, 48(1), 277-280.
Chohan	2013-2014	2016	Poppy seed meal	Aflatoxin B1	25.640	12.820	31.68	8.23	57.51	4	2	50	Asia	Pakistan			ELISA	Chohan, K. A., Awan, F., Ali, M. M., Iqbal, U., & Ijaz, M. (2016). Assessment of Aflatoxin in Dairy Concentrate Feeds, Total Mixed Rations, Silage and Various Feed Ingredients in Pakistan. Pakistan Journal of Zoology, 48(1), 277-280.
Chohan	2013-2014	2016	Rice polish	Aflatoxin B1	8.061	2.850	22.07	15.19	40.15	8	4	50	Asia	Pakistan			ELISA	Chohan, K. A., Awan, F., Ali, M. M., Iqbal, U., & Ijaz, M. (2016). Assessment of Aflatoxin in Dairy Concentrate Feeds, Total Mixed Rations, Silage and Various Feed Ingredients in Pakistan. Pakistan Journal of Zoology, 48(1), 277-280.
Chohan	2013-2014	2016	Sunflower meal	Aflatoxin B1	5.791	2.590	23.86	12.39	39.21	5	2	40	Asia	Pakistan			ELISA	Chohan, K. A., Awan, F., Ali, M. M., Iqbal, U., & Ijaz, M. (2016). Assessment of Aflatoxin in Dairy

																		Concentrate Feeds, Total Mixed Rations, Silage and Various Feed Ingredients in Pakistan. Pakistan Journal of Zoology, 48(1), 277-280.
Chohan	2013-2014	2016	Canola meal	Aflatoxin B1	3.320	1.660	7.08	3.23	21.33	4	1	25	Asia	Pakistan			ELISA	Chohan, K. A., Awan, F., Ali, M. M., Iqbal, U., & Ijaz, M. (2016). Assessment of Aflatoxin in Dairy Concentrate Feeds, Total Mixed Rations, Silage and Various Feed Ingredients in Pakistan. Pakistan Journal of Zoology, 48(1), 277-280.
Chohan	2013-2014	2016	Rape seed meal	Aflatoxin B1	7.540	3.770	8.78	1.58	28.59	4	1	25	Asia	Pakistan			ELISA	Chohan, K. A., Awan, F., Ali, M. M., Iqbal, U., & Ijaz, M. (2016). Assessment of Aflatoxin in Dairy Concentrate Feeds, Total Mixed Rations, Silage and Various Feed Ingredients in Pakistan. Pakistan Journal of Zoology, 48(1), 277-280.
Chohan	2013-2014	2016	Vanda	Aflatoxin B1	8.061	1.140	29.3	5.31	42.39	50	32	64	Asia	Pakistan			ELISA	Chohan, K. A., Awan, F., Ali, M. M., Iqbal, U., & Ijaz, M. (2016). Assessment of Aflatoxin in Dairy Concentrate Feeds, Total Mixed Rations, Silage and Various Feed Ingredients in Pakistan. Pakistan Journal of Zoology, 48(1), 277-280.
Chohan	2013-2014	2016	Silage	Aflatoxin B1	3.741	0.780	9.98	2.51	21.56	23	5	21.74	Asia	Pakistan			ELISA	Chohan, K. A., Awan, F., Ali, M. M., Iqbal, U., & Ijaz, M. (2016). Assessment of Aflatoxin in Dairy Concentrate Feeds, Total Mixed Rations, Silage and Various Feed Ingredients in Pakistan. Pakistan Journal of Zoology, 48(1), 277-280.
Chohan	2013-2014	2016	TMR	Aflatoxin B1	5.280	1.760	21.97	3.02	55.17	9	3	33.33	Asia	Pakistan			ELISA	Chohan, K. A., Awan, F., Ali, M. M., Iqbal, U., & Ijaz, M. (2016). Assessment of Aflatoxin in Dairy Concentrate Feeds, Total Mixed Rations, Silage and Various Feed Ingredients in Pakistan. Pakistan Journal of Zoology, 48(1), 277-280.
Chohan	2013-2014	2016	Hay	Aflatoxin B1	4.327	1.530	4.91	0.56	22.31	8	1	12.5	Asia	Pakistan			ELISA	Chohan, K. A., Awan, F., Ali, M. M., Iqbal, U., & Ijaz, M. (2016). Assessment of Aflatoxin in Dairy Concentrate Feeds, Total Mixed Rations, Silage and Various Feed Ingredients in Pakistan. Pakistan Journal of Zoology, 48(1), 277-280.
Ehsani	2014	2016	Concentrate	Aflatoxin B1	0.042	0.005	9.77	2.08	19.41	70	31	44.3	Asia	Iran	0.08	0.3	HPLC	Ehsani, A., Barani, A., & Nasiri, Z. (2016). Occurrence of aflatoxin B1 contamination in dairy cows feed in Iran. Toxin Reviews, 35(1-2), 54-57. doi:10.3109/15569543.2016.1155622
Ehsani	2014	2016	Corn silage	Aflatoxin B1	0.033	0.004	1098	2.53	18.65	70	18	25.7	Asia	Iran	0.08	0.3	HPLC	Ehsani, A., Barani, A., & Nasiri, Z. (2016). Occurrence of aflatoxin B1 contamination in dairy cows feed in Iran. Toxin Reviews, 35(1-2), 54-57. doi:10.3109/15569543.2016.1155623
Ehsani	2014	2016	Alfalfa hay	Aflatoxin B1	0.033	0.004	10.24	1.87	16.29	70	39	55.7	Asia	Iran	0.08	0.3	HPLC	Ehsani, A., Barani, A., & Nasiri, Z. (2016). Occurrence of aflatoxin B1 contamination in dairy cows feed in Iran. Toxin Reviews, 35(1-2), 54-57. doi:10.3109/15569543.2016.1155624
Ekici	2012	2016	Animal feed	Total Aflatoxins				0	33.9	88	72	81.81	Asia	Turkey	0.03	0.08	ELISA	Ekici, H., Yildirim, E., & Yarsan, E. (2016). The effect of seasonal variations on the occurrence of certain mycotoxins in concentrate feeds for cattle collected from some provinces in Turkey. Turkish Journal of Veterinary & Animal Sciences, 40(3), 298-303. doi:10.3906/vet-1501-71

Ekici	2012	2016	Animal feed	Aflatoxin B1				0	19.24	88	72	81.81	Asia	Turkey	0.01	0.03	ELISA	Ekici, H., Yildirim, E., & Yarsan, E. (2016). The effect of seasonal variations on the occurrence of certain mycotoxins in concentrate feeds for cattle collected from some provinces in Turkey. Turkish Journal of Veterinary & Animal Sciences, 40(3), 298-303. doi:10.3906/vet-1501-71
Ekici	2012	2016	Animal feed	Ochratoxin A				0	79.1	88	84	95.45	Asia	Turkey	0.01	0.04	ELISA	Ekici, H., Yildirim, E., & Yarsan, E. (2016). The effect of seasonal variations on the occurrence of certain mycotoxins in concentrate feeds for cattle collected from some provinces in Turkey. Turkish Journal of Veterinary & Animal Sciences, 40(3), 298-303. doi:10.3906/vet-1501-71
Ekici	2012	2016	Animal feed	Fumonisins				0	3900	88	83	94.31	Asia	Turkey	2	7	ELISA	Ekici, H., Yildirim, E., & Yarsan, E. (2016). The effect of seasonal variations on the occurrence of certain mycotoxins in concentrate feeds for cattle collected from some provinces in Turkey. Turkish Journal of Veterinary & Animal Sciences, 40(3), 298-303. doi:10.3906/vet-1501-71
Hashemi		2016	Corn silage	Aflatoxin B1	9.946	0.980	3.86	0	71.57	103	97	94.17	Asia	Iran	1		ELISA	Hashemi, M. (2016). Aflatoxin B1 levels in feedstuffs from dairy cow farms in south of Iran. Food and Agricultural Immunology, 27(2), 251-258. doi:10.1080/09540105.2015.1086320
Hashemi		2016	Full ration pellet	Aflatoxin B1	5.600	0.700	3.64	0.02	36.07	64	64	100	Asia	Iran	1		ELISA	Hashemi, M. (2016). Aflatoxin B1 levels in feedstuffs from dairy cow farms in south of Iran. Food and Agricultural Immunology, 27(2), 251-258. doi:10.1080/09540105.2015.1086321
Hashemi		2016	Alfalfa hay	Aflatoxin B1	14.527	2.190	6.75	0	66.57	44	43	97.73	Asia	Iran	1		ELISA	Hashemi, M. (2016). Aflatoxin B1 levels in feedstuffs from dairy cow farms in south of Iran. Food and Agricultural Immunology, 27(2), 251-258. doi:10.1080/09540105.2015.1086322
Hashemi		2016	Wheat bran	Aflatoxin B1	8.836	1.380	2.94	0	56.13	41	40	97.56	Asia	Iran	1		ELISA	Hashemi, M. (2016). Aflatoxin B1 levels in feedstuffs from dairy cow farms in south of Iran. Food and Agricultural Immunology, 27(2), 251-258. doi:10.1080/09540105.2015.1086319
Hashemi		2016	Recycled bread	Aflatoxin B1	12.671	2.240	4.32	0	63.11	32	28	87.5	Asia	Iran	1		ELISA	Hashemi, M. (2016). Aflatoxin B1 levels in feedstuffs from dairy cow farms in south of Iran. Food and Agricultural Immunology, 27(2), 251-258. doi:10.1080/09540105.2015.1086324
Hashemi		2016	Crushed yellow corn	Aflatoxin B1	14.640	3.660	9.94	0	45.46	16	14	87.5	Asia	Iran	1		ELISA	Hashemi, M. (2016). Aflatoxin B1 levels in feedstuffs from dairy cow farms in south of Iran. Food and Agricultural Immunology, 27(2), 251-258. doi:10.1080/09540105.2015.1086325
Hashemi		2016	Barley	Aflatoxin B1	1.235	0.330	1.31	0	3.35	14	13	92.81	Asia	Iran	1		ELISA	Hashemi, M. (2016). Aflatoxin B1 levels in feedstuffs from dairy cow farms in south of Iran. Food and Agricultural Immunology, 27(2), 251-258. doi:10.1080/09540105.2015.1086319
Hashemi		2016	Cotton Seed meal	Aflatoxin B1	1.028	0.310	2.13	0.17	3.38	11	11	100	Asia	Iran	1		ELISA	Hashemi, M. (2016). Aflatoxin B1 levels in feedstuffs from dairy cow farms in south of Iran. Food and Agricultural Immunology, 27(2), 251-258. doi:10.1080/09540105.2015.1086319

Hashemi		2016	Beet pulp	Aflatoxin B1	1.535	0.580	1.52	0	4.5	7	6	85.71	Asia	Iran	1		ELISA	Hashemi, M. (2016). Aflatoxin B1 levels in feedstuffs from dairy cow farms in south of Iran. Food and Agricultural Immunology, 27(2), 251-258. doi:10.1080/09540105.2015.1086319
Hashemi		2016	Soya bean meal	Aflatoxin B1	4.101	1.550	6.62	0	11.46	7	5	71.43	Asia	Iran	1		ELISA	Hashemi, M. (2016). Aflatoxin B1 levels in feedstuffs from dairy cow farms in south of Iran. Food and Agricultural Immunology, 27(2), 251-258. doi:10.1080/09540105.2015.1086319
Hashemi		2016	Wheat flour	Aflatoxin B1	0.580	0.290	1.17	0.36	1.71	4	4	100	Asia	Iran	1		ELISA	Hashemi, M. (2016). Aflatoxin B1 levels in feedstuffs from dairy cow farms in south of Iran. Food and Agricultural Immunology, 27(2), 251-258. doi:10.1080/09540105.2015.1086320
Hashemi		2016	Meat powder	Aflatoxin B1	1.480	0.740	2.28	0.91	3.6	4	4	100	Asia	Iran	1		ELISA	Hashemi, M. (2016). Aflatoxin B1 levels in feedstuffs from dairy cow farms in south of Iran. Food and Agricultural Immunology, 27(2), 251-258. doi:10.1080/09540105.2015.1086321
Hashemi		2016	Dairy cattle (Animal feed)	Aflatoxin B1	9.873	0.530	4.12	0	71.57	347	329	94.81	Asia	Iran	1		ELISA	Hashemi, M. (2016). Aflatoxin B1 levels in feedstuffs from dairy cow farms in south of Iran. Food and Agricultural Immunology, 27(2), 251-258. doi:10.1080/09540105.2015.1086319
Iqbal	2012-2013	2016	Cotton Seed meal	Aflatoxin B1	0.450	0.106	4.5	0.09	85.5	18	12	67	Asia	Pakistan	0.03	0.09	HPLC	Iqbal, S. Z., Asi, M. R., Nisar, S., Zia, K. M., Jinap, S., & Malik, N. (2016). A Limited Survey of Aflatoxins and Zearalenone in Feed and Feed Ingredients from Pakistan. J Food Prot, 79(10), 1798-1801. doi:10.4315/0362-028x.jfp-16-091
Iqbal	2012-2013	2016	Cotton Seed meal	Total Aflatoxins	1.200	0.283	6.45		91.5	18	12	67	Asia	Pakistan			HPLC	Iqbal, S. Z., Asi, M. R., Nisar, S., Zia, K. M., Jinap, S., & Malik, N. (2016). A Limited Survey of Aflatoxins and Zearalenone in Feed and Feed Ingredients from Pakistan. J Food Prot, 79(10), 1798-1801. doi:10.4315/0362-028x.jfp-16-091
Iqbal	2012-2013	2016	Soya bean meal	Aflatoxin B1	0.750	0.200	4.9	0.09	105.9	14	9	64	Asia	Pakistan	0.03	0.09	HPLC	Iqbal, S. Z., Asi, M. R., Nisar, S., Zia, K. M., Jinap, S., & Malik, N. (2016). A Limited Survey of Aflatoxins and Zearalenone in Feed and Feed Ingredients from Pakistan. J Food Prot, 79(10), 1798-1801. doi:10.4315/0362-028x.jfp-16-091
Iqbal	2012-2013	2016	Soya bean meal	Total Aflatoxins	0.700	0.187	5.2		135.3	14	9	64	Asia	Pakistan			HPLC	Iqbal, S. Z., Asi, M. R., Nisar, S., Zia, K. M., Jinap, S., & Malik, N. (2016). A Limited Survey of Aflatoxins and Zearalenone in Feed and Feed Ingredients from Pakistan. J Food Prot, 79(10), 1798-1801. doi:10.4315/0362-028x.jfp-16-091
Iqbal	2012-2013	2016	Barley	Aflatoxin B1	1.100	0.267	2.98	0.09	45.2	17	12	70	Asia	Pakistan	0.03	0.09	HPLC	Iqbal, S. Z., Asi, M. R., Nisar, S., Zia, K. M., Jinap, S., & Malik, N. (2016). A Limited Survey of Aflatoxins and Zearalenone in Feed and Feed Ingredients from Pakistan. J Food Prot, 79(10), 1798-1801. doi:10.4315/0362-028x.jfp-16-091
Iqbal	2012-2013	2016	Barley	Total Aflatoxins	0.300	0.073	4.5		50.5	17	12	70	Asia	Pakistan			HPLC	Iqbal, S. Z., Asi, M. R., Nisar, S., Zia, K. M., Jinap, S., & Malik, N. (2016). A Limited Survey of Aflatoxins and Zearalenone in Feed and Feed Ingredients from Pakistan. J Food Prot, 79(10), 1798-1801. doi:10.4315/0362-028x.jfp-16-091

Iqbal	2012-2013	2016	Cotton Seed meal	Zearalenone	0.900	0.212	14.12	0.15	85.54	18	13	67	Asia	Pakistan	0.05	0.15	HPLC	Iqbal, S. Z., Asi, M. R., Nisar, S., Zia, K. M., Jinap, S., & Malik, N. (2016). A Limited Survey of Aflatoxins and Zearalenone in Feed and Feed Ingredients from Pakistan. J Food Prot, 79(10), 1798-1801. doi:10.4315/0362-028x.jfp-16-091
Iqbal	2012-2013	2016	Soya bean meal	Zearalenone	1.780	0.476	18.9	0.15	120.89	14	10	71	Asia	Pakistan	0.05	0.15	HPLC	Iqbal, S. Z., Asi, M. R., Nisar, S., Zia, K. M., Jinap, S., & Malik, N. (2016). A Limited Survey of Aflatoxins and Zearalenone in Feed and Feed Ingredients from Pakistan. J Food Prot, 79(10), 1798-1801. doi:10.4315/0362-028x.jfp-16-091
Iqbal	2012-2013	2016	Barley	Zearalenone	0.980	0.238	8.74	0.15	91.6	17	9	53	Asia	Pakistan	0.05	0.15	HPLC	Iqbal, S. Z., Asi, M. R., Nisar, S., Zia, K. M., Jinap, S., & Malik, N. (2016). A Limited Survey of Aflatoxins and Zearalenone in Feed and Feed Ingredients from Pakistan. J Food Prot, 79(10), 1798-1801. doi:10.4315/0362-028x.jfp-16-091
Iqbal	2012-2013	2016	Crushed corn	Aflatoxin B1	0.980	0.238	5.2	0.09	69.7	17	12	71	Asia	Pakistan	0.03	0.09	HPLC	Iqbal, S. Z., Asi, M. R., Nisar, S., Zia, K. M., Jinap, S., & Malik, N. (2016). A Limited Survey of Aflatoxins and Zearalenone in Feed and Feed Ingredients from Pakistan. J Food Prot, 79(10), 1798-1801. doi:10.4315/0362-028x.jfp-16-092
Iqbal	2012-2013	2016	Crushed wheat	Aflatoxin B1	0.430	0.111	4.25	0.09	55.2	15	8	53	Asia	Pakistan	0.03	0.09	HPLC	Iqbal, S. Z., Asi, M. R., Nisar, S., Zia, K. M., Jinap, S., & Malik, N. (2016). A Limited Survey of Aflatoxins and Zearalenone in Feed and Feed Ingredients from Pakistan. J Food Prot, 79(10), 1798-1801. doi:10.4315/0362-028x.jfp-16-093
Iqbal	2012-2013	2016	Poultry feed 1	Aflatoxin B1	1.400	0.422	6.2	0.09	145.7	11	9	82	Asia	Pakistan	0.03	0.09	HPLC	Iqbal, S. Z., Asi, M. R., Nisar, S., Zia, K. M., Jinap, S., & Malik, N. (2016). A Limited Survey of Aflatoxins and Zearalenone in Feed and Feed Ingredients from Pakistan. J Food Prot, 79(10), 1798-1801. doi:10.4315/0362-028x.jfp-16-094
Iqbal	2012-2013	2016	Poultry feed 2	Aflatoxin B1	0.870	0.241	4.97	0.09	98.3	13	7	54	Asia	Pakistan	0.03	0.09	HPLC	Iqbal, S. Z., Asi, M. R., Nisar, S., Zia, K. M., Jinap, S., & Malik, N. (2016). A Limited Survey of Aflatoxins and Zearalenone in Feed and Feed Ingredients from Pakistan. J Food Prot, 79(10), 1798-1801. doi:10.4315/0362-028x.jfp-16-095
Iqbal	2012-2013	2016	Crushed corn	Total Aflatoxins	0.980	0.238	7.8		85.4	17	12	71	Asia	Pakistan			HPLC	Iqbal, S. Z., Asi, M. R., Nisar, S., Zia, K. M., Jinap, S., & Malik, N. (2016). A Limited Survey of Aflatoxins and Zearalenone in Feed and Feed Ingredients from Pakistan. J Food Prot, 79(10), 1798-1801. doi:10.4315/0362-028x.jfp-16-092
Iqbal	2012-2013	2016	Crushed wheat	Total Aflatoxins	0.830	0.214	6.73		55.2	15	8	53	Asia	Pakistan			HPLC	Iqbal, S. Z., Asi, M. R., Nisar, S., Zia, K. M., Jinap, S., & Malik, N. (2016). A Limited Survey of Aflatoxins and Zearalenone in Feed and Feed Ingredients from Pakistan. J Food Prot, 79(10), 1798-1801. doi:10.4315/0362-028x.jfp-16-093
Iqbal	2012-2013	2016	Poultry feed 1	Total Aflatoxins	1.400	0.422	9.3		165.5	11	9	82	Asia	Pakistan			HPLC	Iqbal, S. Z., Asi, M. R., Nisar, S., Zia, K. M., Jinap, S., & Malik, N. (2016). A Limited Survey of Aflatoxins and Zearalenone in Feed and Feed Ingredients from Pakistan. J Food Prot, 79(10), 1798-1801. doi:10.4315/0362-028x.jfp-16-094

Iqbal	2012-2013	2016	Poultry feed 2	Total Aflatoxins	1.100	0.305	7.89		103.1	13	7	54	Asia	Pakistan			HPLC	Iqbal, S. Z., Asi, M. R., Nisar, S., Zia, K. M., Jinap, S., & Malik, N. (2016). A Limited Survey of Aflatoxins and Zearalenone in Feed and Feed Ingredients from Pakistan. J Food Prot, 79(10), 1798-1801. doi:10.4315/0362-028x.jfp-16-095
Iqbal	2012-2013	2016	Crushed corn	Zearalenone	0.290	0.070	9.87	0.15	69.9	17	14	82	Asia	Pakistan	0.05	0.15	HPLC	Iqbal, S. Z., Asi, M. R., Nisar, S., Zia, K. M., Jinap, S., & Malik, N. (2016). A Limited Survey of Aflatoxins and Zearalenone in Feed and Feed Ingredients from Pakistan. J Food Prot, 79(10), 1798-1801. doi:10.4315/0362-028x.jfp-16-092
Iqbal	2012-2013	2016	Crushed wheat	Zearalenone	1.600	0.413	18.64	0.15	145.3	15	10	67	Asia	Pakistan	0.05	0.15	HPLC	Iqbal, S. Z., Asi, M. R., Nisar, S., Zia, K. M., Jinap, S., & Malik, N. (2016). A Limited Survey of Aflatoxins and Zearalenone in Feed and Feed Ingredients from Pakistan. J Food Prot, 79(10), 1798-1801. doi:10.4315/0362-028x.jfp-16-093
Iqbal	2012-2013	2016	Poultry feed 1	Zearalenone	0.700	0.211	15.8	0.15	125.2	11	9	82	Asia	Pakistan	0.05	0.15	HPLC	Iqbal, S. Z., Asi, M. R., Nisar, S., Zia, K. M., Jinap, S., & Malik, N. (2016). A Limited Survey of Aflatoxins and Zearalenone in Feed and Feed Ingredients from Pakistan. J Food Prot, 79(10), 1798-1801. doi:10.4315/0362-028x.jfp-16-094
Iqbal	2012-2013	2016	Poultry feed 2	Zearalenone	0.880	0.244	19.45	0.15	118.42	13	10	77	Asia	Pakistan	0.05	0.15	HPLC	Iqbal, S. Z., Asi, M. R., Nisar, S., Zia, K. M., Jinap, S., & Malik, N. (2016). A Limited Survey of Aflatoxins and Zearalenone in Feed and Feed Ingredients from Pakistan. J Food Prot, 79(10), 1798-1801. doi:10.4315/0362-028x.jfp-16-095
Sahin	2012-2015	2016	Dairy cattle (Animal feed)	Aflatoxin B1	0.120	0.014	2.25	0.278	6.89	76	21	26.3	Asia	Turkey	0.054	0.181	HPLC	Sahin, H. Z., Celik, M., Kotay, S., & Kabak, B. (2016). Aflatoxins in dairy cow feed, raw milk and milk products from Turkey. Food Addit Contam Part B Surveill, 9(2), 152-158. doi:10.1080/19393210.2016.1152599
Sahin	2012-2015	2016	Dairy cattle (Animal feed)	Aflatoxin B2	0.099	0.011	0.231	0.081	0.752	76	18	23.7	Asia	Turkey	0.046	0.153	HPLC	Sahin, H. Z., Celik, M., Kotay, S., & Kabak, B. (2016). Aflatoxins in dairy cow feed, raw milk and milk products from Turkey. Food Addit Contam Part B Surveill, 9(2), 152-158. doi:10.1080/19393210.2016.1152599
Sahin	2012-2015	2016	Dairy cattle (Animal feed)	Aflatoxin G1	0.021	0.002	0.334	0.207	0.788	76	17	22.4	Asia	Turkey	0.059	0.197	HPLC	Sahin, H. Z., Celik, M., Kotay, S., & Kabak, B. (2016). Aflatoxins in dairy cow feed, raw milk and milk products from Turkey. Food Addit Contam Part B Surveill, 9(2), 152-158. doi:10.1080/19393210.2016.1152599
Sahin	2012-2015	2016	Dairy cattle (Animal feed)	Aflatoxin G2						76	0	0	Asia	Turkey	0.05	0.168	HPLC	Sahin, H. Z., Celik, M., Kotay, S., & Kabak, B. (2016). Aflatoxins in dairy cow feed, raw milk and milk products from Turkey. Food Addit Contam Part B Surveill, 9(2), 152-158. doi:10.1080/19393210.2016.1152600
Sahin	2012-2015	2016	Dairy cattle (Animal feed)	Total Aflatoxins			2.74	0.278	8.43	76	20	26.3	Asia	Turkey			HPLC	Sahin, H. Z., Celik, M., Kotay, S., & Kabak, B. (2016). Aflatoxins in dairy cow feed, raw milk and milk products from Turkey. Food Addit Contam Part B Surveill, 9(2), 152-158. doi:10.1080/19393210.2016.1152599

Sifou	2013-2014	2016	Poultry (Animal Feed)	Ochratoxin A	2.400	0.305	7.1		26.8	62	19	30.6	Asia	Morocco		0.15	HPLC	Sifou, A., Mahnine, N., Manyes, L., Adlouni, C. E., Azzouzi, M. E., & Zinedine, A. (2016). Determination of Ochratoxin A in poultry feeds available in Rabat area (Morocco) by High Performance Liquid Chromatography. Journal of Materials and Environmental Science, 7(6), 2229-2234. Retrieved from https://www.scopus.com/inward/record.uri?eid=2-s2.0-84976494160&partnerID=40&md5=83c40aeaa17544064a053983f2dab6fa
Abdallah	2014-2015	2017	Animal feed	Aflatoxin B1			5.3		0.72	77	3	4	Africa	Egypt	0.72	2.4	LC-MS/MS	Abdallah, M. F., Girgin, G., Baydar, T., Krska, R., & Sulyok, M. (2017). Occurrence of multiple mycotoxins and other fungal metabolites in animal feed and maize samples from Egypt using LC-MS/MS. J Sci Food Agric, 97(13), 4419-4428. doi:10.1002/jsfa.8293
Abdallah	2014-2015	2017	Animal feed	Alpha Zearalenone			7.3		8	77	5	6	Africa	Egypt	1.3	4.5	LC-MS/MS	Abdallah, M. F., Girgin, G., Baydar, T., Krska, R., & Sulyok, M. (2017). Occurrence of multiple mycotoxins and other fungal metabolites in animal feed and maize samples from Egypt using LC-MS/MS. J Sci Food Agric, 97(13), 4419-4428. doi:10.1002/jsfa.8293
Abdallah	2014-2015	2017	Animal feed	Beta Zearalenone			3.9		60	77	28	36	Africa	Egypt	1.2	3.5	LC-MS/MS	Abdallah, M. F., Girgin, G., Baydar, T., Krska, R., & Sulyok, M. (2017). Occurrence of multiple mycotoxins and other fungal metabolites in animal feed and maize samples from Egypt using LC-MS/MS. J Sci Food Agric, 97(13), 4419-4428. doi:10.1002/jsfa.8293
Abdallah	2014-2015	2017	Animal feed	Deoxynivalenol			171		1516	77	55	71	Africa	Egypt	9.5	31	LC-MS/MS	Abdallah, M. F., Girgin, G., Baydar, T., Krska, R., & Sulyok, M. (2017). Occurrence of multiple mycotoxins and other fungal metabolites in animal feed and maize samples from Egypt using LC-MS/MS. J Sci Food Agric, 97(13), 4419-4428. doi:10.1002/jsfa.8293
Abdallah	2014-2015	2017	Animal feed	Fumonisin B1			459		2409	77	59	77	Africa	Egypt	2.6	8.5	LC-MS/MS	Abdallah, M. F., Girgin, G., Baydar, T., Krska, R., & Sulyok, M. (2017). Occurrence of multiple mycotoxins and other fungal metabolites in animal feed and maize samples from Egypt using LC-MS/MS. J Sci Food Agric, 97(13), 4419-4428. doi:10.1002/jsfa.8293
Abdallah	2014-2015	2017	Animal feed	Fumonisin B2			55.4		260	77	53	69	Africa	Egypt	1	3.3	LC-MS/MS	Abdallah, M. F., Girgin, G., Baydar, T., Krska, R., & Sulyok, M. (2017). Occurrence of multiple mycotoxins and other fungal metabolites in animal feed and maize samples from Egypt using LC-MS/MS. J Sci Food Agric, 97(13), 4419-4428. doi:10.1002/jsfa.8293
Abdallah	2014-2015	2017	Animal feed	Fumonisin B3			106		310	77	42	55	Africa	Egypt	3.8	11	LC-MS/MS	Abdallah, M. F., Girgin, G., Baydar, T., Krska, R., & Sulyok, M. (2017). Occurrence of multiple mycotoxins and other fungal metabolites in animal feed and maize samples from Egypt using LC-

															MS/MS. J Sci Food Agric, 97(13), 4419-4428. doi:10.1002/jsfa.8293
Abdallah	2014-2015	2017	Animal feed	HT-2 toxin		32.3	77	10	13	Africa	Egypt	1.7	5.7	LC-MS/MS	Abdallah, M. F., Girgin, G., Baydar, T., Krska, R., & Sulyok, M. (2017). Occurrence of multiple mycotoxins and other fungal metabolites in animal feed and maize samples from Egypt using LC-MS/MS. J Sci Food Agric, 97(13), 4419-4428. doi:10.1002/jsfa.8293
Abdallah	2014-2015	2017	Animal feed	T-2 toxin	5.8	39.5	77	19	25	Africa	Egypt	1.05	3.5	LC-MS/MS	Abdallah, M. F., Girgin, G., Baydar, T., Krska, R., & Sulyok, M. (2017). Occurrence of multiple mycotoxins and other fungal metabolites in animal feed and maize samples from Egypt using LC-MS/MS. J Sci Food Agric, 97(13), 4419-4428. doi:10.1002/jsfa.8293
Abdallah	2014-2015	2017	Animal feed	Zearalenone	33.2	791	77	71	92	Africa	Egypt	0.64	2.1	LC-MS/MS	Abdallah, M. F., Girgin, G., Baydar, T., Krska, R., & Sulyok, M. (2017). Occurrence of multiple mycotoxins and other fungal metabolites in animal feed and maize samples from Egypt using LC-MS/MS. J Sci Food Agric, 97(13), 4419-4428. doi:10.1002/jsfa.8293
Abdallah	2014-2015	2017	Maize	Aflatoxin B1	4.81	197.5	79	13	16	Africa	Egypt	0.3	0.98	LC-MS/MS	Abdallah, M. F., Girgin, G., Baydar, T., Krska, R., & Sulyok, M. (2017). Occurrence of multiple mycotoxins and other fungal metabolites in animal feed and maize samples from Egypt using LC-MS/MS. J Sci Food Agric, 97(13), 4419-4428. doi:10.1002/jsfa.8293
Abdallah	2014-2015	2017	Maize	Aflatoxin B2	7.65	9.8	79	4	5	Africa	Egypt	0.42	1.4	LC-MS/MS	Abdallah, M. F., Girgin, G., Baydar, T., Krska, R., & Sulyok, M. (2017). Occurrence of multiple mycotoxins and other fungal metabolites in animal feed and maize samples from Egypt using LC-MS/MS. J Sci Food Agric, 97(13), 4419-4428. doi:10.1002/jsfa.8293
Abdallah	2014-2015	2017	Maize	Aflatoxin M1	1.2	2.7	79	4	5	Africa	Egypt	0.1	0.32	LC-MS/MS	Abdallah, M. F., Girgin, G., Baydar, T., Krska, R., & Sulyok, M. (2017). Occurrence of multiple mycotoxins and other fungal metabolites in animal feed and maize samples from Egypt using LC-MS/MS. J Sci Food Agric, 97(13), 4419-4428. doi:10.1002/jsfa.8293
Abdallah	2014-2015	2017	Maize	Deoxynivalenol	311	807	79	6	8	Africa	Egypt	26	86	LC-MS/MS	Abdallah, M. F., Girgin, G., Baydar, T., Krska, R., & Sulyok, M. (2017). Occurrence of multiple mycotoxins and other fungal metabolites in animal feed and maize samples from Egypt using LC-MS/MS. J Sci Food Agric, 97(13), 4419-4428. doi:10.1002/jsfa.8293
Abdallah	2014-2015	2017	Maize	Fumonisin B1	68	2453	79	40	51	Africa	Egypt	1	3.3	LC-MS/MS	Abdallah, M. F., Girgin, G., Baydar, T., Krska, R., & Sulyok, M. (2017). Occurrence of multiple mycotoxins and other fungal metabolites in animal feed and maize samples from Egypt using LC-MS/MS. J Sci Food Agric, 97(13), 4419-4428. doi:10.1002/jsfa.8293

Abdallah	2014-2015	2017	Maize	Fumonisin B2			4.7		386	79	14	18	Africa	Egypt	1.3	4.3	LC-MS/MS	Abdallah, M. F., Girgin, G., Baydar, T., Krska, R., & Sulyok, M. (2017). Occurrence of multiple mycotoxins and other fungal metabolites in animal feed and maize samples from Egypt using LC-MS/MS. J Sci Food Agric, 97(13), 4419-4428. doi:10.1002/jsfa.8293
Abdallah	2014-2015	2017	Maize	Fumonisin B3			16.8		286	79	6	8	Africa	Egypt	1.5	4.9	LC-MS/MS	Abdallah, M. F., Girgin, G., Baydar, T., Krska, R., & Sulyok, M. (2017). Occurrence of multiple mycotoxins and other fungal metabolites in animal feed and maize samples from Egypt using LC-MS/MS. J Sci Food Agric, 97(13), 4419-4428. doi:10.1002/jsfa.8293
Abdallah	2014-2015	2017	Maize	Ochratoxin A					11	79	2	3	Africa	Egypt	2.8	9.4	LC-MS/MS	Abdallah, M. F., Girgin, G., Baydar, T., Krska, R., & Sulyok, M. (2017). Occurrence of multiple mycotoxins and other fungal metabolites in animal feed and maize samples from Egypt using LC-MS/MS. J Sci Food Agric, 97(13), 4419-4428. doi:10.1002/jsfa.8293
Abdallah	2014-2015	2017	Maize	Zearalenone			3.4		184	79	10	13	Africa	Egypt	0.46	1.5	LC-MS/MS	Abdallah, M. F., Girgin, G., Baydar, T., Krska, R., & Sulyok, M. (2017). Occurrence of multiple mycotoxins and other fungal metabolites in animal feed and maize samples from Egypt using LC-MS/MS. J Sci Food Agric, 97(13), 4419-4428. doi:10.1002/jsfa.8293
Abudabos		2017	Distiller’s dried grains with solubles	Aflatoxin B1	2.180	0.178	5.8	1	9.9	150	21	14	Asia	Saudi Arabia			HPLC	Abudabos, A. M., Al-Atiyat, R. M., & Khan, R. U. (2017). A survey of mycotoxin contamination and chemical composition of distiller's dried grains with solubles (DDGS) imported from the USA into Saudi Arabia. Environ Sci Pollut Res Int, 24(18), 15401-15405. doi:10.1007/s11356-017-9130-2
Abudabos		2017	Distiller’s dried grains with solubles	Aflatoxin B2	0.090	0.007	0.5	0.3	0.6	150	9	6	Asia	Saudi Arabia			HPLC	Abudabos, A. M., Al-Atiyat, R. M., & Khan, R. U. (2017). A survey of mycotoxin contamination and chemical composition of distiller's dried grains with solubles (DDGS) imported from the USA into Saudi Arabia. Environ Sci Pollut Res Int, 24(18), 15401-15405. doi:10.1007/s11356-017-9130-2
Abudabos		2017	Distiller’s dried grains with solubles	Aflatoxin G1	0.150	0.012	0.5	0.4	0.7	150	4	2.7	Asia	Saudi Arabia			HPLC	Abudabos, A. M., Al-Atiyat, R. M., & Khan, R. U. (2017). A survey of mycotoxin contamination and chemical composition of distiller's dried grains with solubles (DDGS) imported from the USA into Saudi Arabia. Environ Sci Pollut Res Int, 24(18), 15401-15405. doi:10.1007/s11356-017-9130-2
Abudabos		2017	Distiller’s dried grains with solubles	Aflatoxin G2	0.260	0.021	0.8	0.5	1.1	150	6	4	Asia	Saudi Arabia			HPLC	Abudabos, A. M., Al-Atiyat, R. M., & Khan, R. U. (2017). A survey of mycotoxin contamination and chemical composition of distiller's dried grains with solubles (DDGS) imported from the USA into Saudi Arabia. Environ Sci Pollut Res Int, 24(18), 15401-15405. doi:10.1007/s11356-017-9130-2
Abudabos		2017	Distiller’s dried grains	Total Aflatoxins	2.600	0.212	6.3	1	11.3	150	21	14	Asia	Saudi Arabia			HPLC	Abudabos, A. M., Al-Atiyat, R. M., & Khan, R. U. (2017). A survey of mycotoxin contamination and chemical composition of distiller's dried grains with

			with soluble														solubles (DDGS) imported from the USA into Saudi Arabia. Environ Sci Pollut Res Int, 24(18), 15401-15405. doi:10.1007/s11356-017-9130-2
Abudabos		2017	Distiller's dried grains with solubles	Deoxynivalenol	2.240	0.183	3000	800	8100	150	43	28.7	Asia	Saudi Arabia		HPLC	Abudabos, A. M., Al-Atiyat, R. M., & Khan, R. U. (2017). A survey of mycotoxin contamination and chemical composition of distiller's dried grains with solubles (DDGS) imported from the USA into Saudi Arabia. Environ Sci Pollut Res Int, 24(18), 15401-15405. doi:10.1007/s11356-017-9130-2
Abudabos		2017	Distiller's dried grains with solubles	Fumonisin B1	0.780	0.064	1640	430	3640	150	38	25.3	Asia	Saudi Arabia		HPLC	Abudabos, A. M., Al-Atiyat, R. M., & Khan, R. U. (2017). A survey of mycotoxin contamination and chemical composition of distiller's dried grains with solubles (DDGS) imported from the USA into Saudi Arabia. Environ Sci Pollut Res Int, 24(18), 15401-15405. doi:10.1007/s11356-017-9130-2
Abudabos		2017	Distiller's dried grains with solubles	Fumonisin B2	0.500	0.041	590	210	2100	150	35	23.3	Asia	Saudi Arabia		HPLC	Abudabos, A. M., Al-Atiyat, R. M., & Khan, R. U. (2017). A survey of mycotoxin contamination and chemical composition of distiller's dried grains with solubles (DDGS) imported from the USA into Saudi Arabia. Environ Sci Pollut Res Int, 24(18), 15401-15405. doi:10.1007/s11356-017-9130-2
Abudabos		2017	Distiller's dried grains with solubles	Fumonisin B3	0.160	0.013	360	210	650	150	9	6	Asia	Saudi Arabia		HPLC	Abudabos, A. M., Al-Atiyat, R. M., & Khan, R. U. (2017). A survey of mycotoxin contamination and chemical composition of distiller's dried grains with solubles (DDGS) imported from the USA into Saudi Arabia. Environ Sci Pollut Res Int, 24(18), 15401-15405. doi:10.1007/s11356-017-9130-2
Abudabos		2017	Distiller's dried grains with solubles	Fumonisins	0.550	0.045	1010	210	2200	150	38	25.3	Asia	Saudi Arabia		HPLC	Abudabos, A. M., Al-Atiyat, R. M., & Khan, R. U. (2017). A survey of mycotoxin contamination and chemical composition of distiller's dried grains with solubles (DDGS) imported from the USA into Saudi Arabia. Environ Sci Pollut Res Int, 24(18), 15401-15405. doi:10.1007/s11356-017-9130-2
Abudabos		2017	Distiller's dried grains with solubles	Zearalenone	109.650	8.953	167.6	33	501	150	52	34.7	Asia	Saudi Arabia		HPLC	Abudabos, A. M., Al-Atiyat, R. M., & Khan, R. U. (2017). A survey of mycotoxin contamination and chemical composition of distiller's dried grains with solubles (DDGS) imported from the USA into Saudi Arabia. Environ Sci Pollut Res Int, 24(18), 15401-15405. doi:10.1007/s11356-017-9130-2
Ismail	2014-2015	2017	Commercial feed	Aflatoxin B1			4.92	1.04	9.76	72	22	30.5	Asia	Pakistan		ELISA	Ismail, A., Riaz, M., Akhtar, S., Yoo, S. H., Park, S., Abid, M., . . . Ahmad, Z. (2017). Seasonal variation of aflatoxin B-1 content in dairy feed. Journal of Animal and Feed Sciences, 26(1), 33-37. doi:10.22358/jafs/69008/2017
Ismail	2014-2015	2017	Fresh fodder	Aflatoxin B1			3.04	0.64	4.6	72	2	2.8	Asia	Pakistan		ELISA	Ismail, A., Riaz, M., Akhtar, S., Yoo, S. H., Park, S., Abid, M., . . . Ahmad, Z. (2017). Seasonal variation of aflatoxin B-1 content in dairy feed. Journal of Animal and Feed Sciences, 26(1), 33-37. doi:10.22358/jafs/69008/2017

Ismail	2014-2015	2017	Leftover Bread Sample	Aflatoxin B1			6.72	3.96	11.34	72	64	88.9	Asia	Pakistan			ELISA	Ismail, A., Riaz, M., Akhtar, S., Yoo, S. H., Park, S., Abid, M., . . . Ahmad, Z. (2017). Seasonal variation of aflatoxin B-1 content in dairy feed. Journal of Animal and Feed Sciences, 26(1), 33-37. doi:10.22358/jafs/69008/2017
Jedidi	2011	2017	Harvest time Maize	Aflatoxin B1	51.545	16.300	18.98			10	4	40	Africa	Tunisia	0.2	0.6	HPLC	Jedidi, I., Cruz, A., Gonzalez-Jaen, M. T., & Said, S. (2017). Aflatoxins and ochratoxin A and their Aspergillus causal species in Tunisian cereals. Food Additives & Contaminants Part B-Surveillance, 10(1), 51-58. doi:10.1080/19393210.2016.1247917
Jedidi	2011	2017	Harvest time Maize	Aflatoxin B2	5.439	1.720	1.93			10	4	40	Africa	Tunisia	0.04	0.12	HPLC	Jedidi, I., Cruz, A., Gonzalez-Jaen, M. T., & Said, S. (2017). Aflatoxins and ochratoxin A and their Aspergillus causal species in Tunisian cereals. Food Additives & Contaminants Part B-Surveillance, 10(1), 51-58. doi:10.1080/19393210.2016.1247917
Jedidi	2011	2017	Harvest time Maize	Aflatoxin G1	203.967	64.500	76.01			10	9	90	Africa	Tunisia	0.02	0.06	HPLC	Jedidi, I., Cruz, A., Gonzalez-Jaen, M. T., & Said, S. (2017). Aflatoxins and ochratoxin A and their Aspergillus causal species in Tunisian cereals. Food Additives & Contaminants Part B-Surveillance, 10(1), 51-58. doi:10.1080/19393210.2016.1247917
Jedidi	2011	2017	Harvest time Maize	Aflatoxin G2	41.489	13.120	14.56			10	8	80	Africa	Tunisia	0.01	0.03	HPLC	Jedidi, I., Cruz, A., Gonzalez-Jaen, M. T., & Said, S. (2017). Aflatoxins and ochratoxin A and their Aspergillus causal species in Tunisian cereals. Food Additives & Contaminants Part B-Surveillance, 10(1), 51-58. doi:10.1080/19393210.2016.1247917
Jedidi	2011	2017	Harvest time Maize	Total Aflatoxins	302.409	95.630	111.47			10	9	90	Africa	Tunisia			HPLC	Jedidi, I., Cruz, A., Gonzalez-Jaen, M. T., & Said, S. (2017). Aflatoxins and ochratoxin A and their Aspergillus causal species in Tunisian cereals. Food Additives & Contaminants Part B-Surveillance, 10(1), 51-58. doi:10.1080/19393210.2016.1247917
Jedidi	2011	2017	Postharvest time Maize	Aflatoxin B1	15.422	4.650	4.65			11	1	9.09	Africa	Tunisia	0.2	0.6	HPLC	Jedidi, I., Cruz, A., Gonzalez-Jaen, M. T., & Said, S. (2017). Aflatoxins and ochratoxin A and their Aspergillus causal species in Tunisian cereals. Food Additives & Contaminants Part B-Surveillance, 10(1), 51-58. doi:10.1080/19393210.2016.1247917
Jedidi	2011	2017	Postharvest time Maize	Aflatoxin B2	0.099	0.030	0.03			11	1	9.09	Africa	Tunisia	0.04	0.12	HPLC	Jedidi, I., Cruz, A., Gonzalez-Jaen, M. T., & Said, S. (2017). Aflatoxins and ochratoxin A and their Aspergillus causal species in Tunisian cereals. Food Additives & Contaminants Part B-Surveillance, 10(1), 51-58. doi:10.1080/19393210.2016.1247917
Jedidi	2011	2017	Postharvest time Maize	Aflatoxin G1	13.797	4.160	6.34			11	9	81.82	Africa	Tunisia	0.02	0.06	HPLC	Jedidi, I., Cruz, A., Gonzalez-Jaen, M. T., & Said, S. (2017). Aflatoxins and ochratoxin A and their Aspergillus causal species in Tunisian cereals. Food Additives & Contaminants Part B-Surveillance, 10(1), 51-58. doi:10.1080/19393210.2016.1247917
Jedidi	2011	2017	Postharvest time Maize	Aflatoxin G2	0.033	0.010	0.06			11	7	72.73	Africa	Tunisia	0.01	0.03	HPLC	Jedidi, I., Cruz, A., Gonzalez-Jaen, M. T., & Said, S. (2017). Aflatoxins and ochratoxin A and their Aspergillus causal species in Tunisian cereals. Food Additives & Contaminants Part B-Surveillance, 10(1), 51-58. doi:10.1080/19393210.2016.1247917

Jedidi	2011	2017	Postharvest time Maize	Total Aflatoxins	29.319	8.840	11.08			11	9	81.82	Africa	Tunisia			HPLC	Jedidi, I., Cruz, A., Gonzalez-Jaen, M. T., & Said, S. (2017). Aflatoxins and ochratoxin A and their Aspergillus causal species in Tunisian cereals. Food Additives & Contaminants Part B-Surveillance, 10(1), 51-58. doi:10.1080/19393210.2016.1247917
Yalcin	2014	2017	Poultry Feed	Aflatoxin B1						73	23	32	Asia	Turkey	0.3	1	LC-MC	YALCIN, N. F., ISIK, M. K., AVCI, T., OGUZ, H. & YURDUSEVEN, T. 2017. Investigation of mycotoxin residues in poultry feeds by LC MS/MS method. Ankara Universitesi Veteriner Fakultesi Dergisi, 64, 111-116.
Yalcin	2014	2017	Poultry Feed	Ochratoxin A						73	34	47	Asia	Turkey	0.5	1.25	LC-MC	YALCIN, N. F., ISIK, M. K., AVCI, T., OGUZ, H. & YURDUSEVEN, T. 2017. Investigation of mycotoxin residues in poultry feeds by LC MS/MS method. Ankara Universitesi Veteriner Fakultesi Dergisi, 64, 111-116.
Yalcin	2014	2017	Poultry Feed	Fumonisins						73	66	90	Asia	Turkey	30	100	LC-MC	YALCIN, N. F., ISIK, M. K., AVCI, T., OGUZ, H. & YURDUSEVEN, T. 2017. Investigation of mycotoxin residues in poultry feeds by LC MS/MS method. Ankara Universitesi Veteriner Fakultesi Dergisi, 64, 111-116.
Yalcin	2014	2017	Poultry Feed	Deoxynivalenol						73	7	10	Asia	Turkey	25	75	LC-MC	YALCIN, N. F., ISIK, M. K., AVCI, T., OGUZ, H. & YURDUSEVEN, T. 2017. Investigation of mycotoxin residues in poultry feeds by LC MS/MS method. Ankara Universitesi Veteriner Fakultesi Dergisi, 64, 111-116.
Yalcin	2014	2017	Poultry Feed	Zearalenone						73	28	38	Asia	Turkey	5	15	LC-MC	YALCIN, N. F., ISIK, M. K., AVCI, T., OGUZ, H. & YURDUSEVEN, T. 2017. Investigation of mycotoxin residues in poultry feeds by LC MS/MS method. Ankara Universitesi Veteriner Fakultesi Dergisi, 64, 111-116.
Yalcin	2014	2017	Poultry Feed	T-2 toxin						73	0	0	Asia	Turkey	30	100	LC-MC	YALCIN, N. F., ISIK, M. K., AVCI, T., OGUZ, H. & YURDUSEVEN, T. 2017. Investigation of mycotoxin residues in poultry feeds by LC MS/MS method. Ankara Universitesi Veteriner Fakultesi Dergisi, 64, 111-116.
Yalcin	2014	2017	Poultry Feed	HT-2 toxin						73	2	3	Asia	Turkey	30	100	LC-MC	YALCIN, N. F., ISIK, M. K., AVCI, T., OGUZ, H. & YURDUSEVEN, T. 2017. Investigation of mycotoxin residues in poultry feeds by LC MS/MS method. Ankara Universitesi Veteriner Fakultesi Dergisi, 64, 111-116.
Yildirim	2012-203	2018	Feed	Total Aflatoxins	7.010	0.565	6.43	0.2	28.8	154	154	100	Asia	Turkey			ELISA	YILDIRIM, E., MACUN, H. C., YALÇINKAYA, İ., ŞAHINDOKUYUCU KOCASARI, F. & EKICI, H. 2018. Survey of aflatoxin residue in feed and milk samples in Kırıkkale province, Turkey. Ankara Universitesi Veteriner Fakultesi Dergisi, 65, 199-204.
Al Khalaileh	2016	2018	Feed	Ochratoxin A	0.260	0.042	2.9	1.72	3.7	39	15	38	Asia	Jordan			ELISA	AL KHALAILEH, N. I. 2018. Prevalence of Ochratoxin A in Poultry Feed and Meat from Jordan. Pak J Biol Sci, 21, 239-244.
Al Khalaileh	2016	2018	Corn	Ochratoxin A	0.320	0.092	2.35	1.54	3.18	12	6	50	Asia	Jordan			ELISA	AL KHALAILEH, N. I. 2018. Prevalence of Ochratoxin A in Poultry Feed and Meat from Jordan. Pak J Biol Sci, 21, 239-244.

Al Khalaileh	2016	2018	Feed + Sun	Ochratoxin A	0.590	0.241	10.3	2.1	27.6	6	6	100	Asia	Jordan			ELISA	AL KHALAILEH, N. I. 2018. Prevalence of Ochratoxin A in Poultry Feed and Meat from Jordan. Pak J Biol Sci, 21, 239-244.
Zebiri	2013	2019	Wheat & Barn	Ochratoxin A	5.800	1.077	6.95	0.16	34.75	29	22	76	Africa	Algeria	0.1	0.5	HPLC-FLD	ZEBIRI, S., MOKRANE, S., VERHEECKE-VAESSEN, C., CHOQUE, E., REGHIOUI, H., SABAOU, N., MATHIEU, F. & RIBA, A. 2019. Occurrence of ochratoxin A in Algerian wheat and its milling derivatives. Toxin Reviews, 38, 206-211.
Zebiri	2013	2019	Wheat & Barn	Ochratoxin A	6.700	1.858	6.77	0.36	18.18	13	13	100	Africa	Algeria	0.15	0.4	HPLC-FLD	ZEBIRI, S., MOKRANE, S., VERHEECKE-VAESSEN, C., CHOQUE, E., REGHIOUI, H., SABAOU, N., MATHIEU, F. & RIBA, A. 2019. Occurrence of ochratoxin A in Algerian wheat and its milling derivatives. Toxin Reviews, 38, 206-211.
Hassan	0	2019	Mixed cereals	Zearalenone			25		75	16	14	88	Asia	Qatar	1.75	0	ELISA	UL HASSAN, Z., AL THANI, R., BALMAS, V., MIGHELI, Q. & JAOUA, S. 2019. Prevalence of Fusarium fungi and their toxins in marketed feed. Food Control, 104, 224-230.
Hassan	0	2019	Mixed cereals	Deoxynivalenol			158		367	16	10	63	Asia	Qatar	18.5	0	ELISA	UL HASSAN, Z., AL THANI, R., BALMAS, V., MIGHELI, Q. & JAOUA, S. 2019. Prevalence of Fusarium fungi and their toxins in marketed feed. Food Control, 104, 224-230.
Hassan	0	2019	Mixed cereals	Fumonisins			930		2360	16	16	100	Asia	Qatar	25	0	ELISA	UL HASSAN, Z., AL THANI, R., BALMAS, V., MIGHELI, Q. & JAOUA, S. 2019. Prevalence of Fusarium fungi and their toxins in marketed feed. Food Control, 104, 224-230.
Hassan	0	2019	Maize	Zearalenone			17		53	10	5	50	Asia	Qatar	1.75	0	ELISA	UL HASSAN, Z., AL THANI, R., BALMAS, V., MIGHELI, Q. & JAOUA, S. 2019. Prevalence of Fusarium fungi and their toxins in marketed feed. Food Control, 104, 224-230.
Hassan	0	2019	Maize	Deoxynivalenol			60		86	10	3	30	Asia	Qatar	18.5	0	ELISA	UL HASSAN, Z., AL THANI, R., BALMAS, V., MIGHELI, Q. & JAOUA, S. 2019. Prevalence of Fusarium fungi and their toxins in marketed feed. Food Control, 104, 224-230.
Hassan	0	2019	Maize	Fumonisins			1026		2640	10	8	80	Asia	Qatar	25	0	ELISA	UL HASSAN, Z., AL THANI, R., BALMAS, V., MIGHELI, Q. & JAOUA, S. 2019. Prevalence of Fusarium fungi and their toxins in marketed feed. Food Control, 104, 224-230.
Hassan	0	2019	Wheat	Zearalenone			8		11	10	4	40	Asia	Qatar	1.75	0	ELISA	UL HASSAN, Z., AL THANI, R., BALMAS, V., MIGHELI, Q. & JAOUA, S. 2019. Prevalence of Fusarium fungi and their toxins in marketed feed. Food Control, 104, 224-230.
Hassan	0	2019	Wheat	Deoxynivalenol			122		222	10	4	40	Asia	Qatar	18.5	0	ELISA	UL HASSAN, Z., AL THANI, R., BALMAS, V., MIGHELI, Q. & JAOUA, S. 2019. Prevalence of Fusarium fungi and their toxins in marketed feed. Food Control, 104, 224-230.
Hassan	0	2019	Wheat	Fumonisins			497		760	10	8	80	Asia	Qatar	25	0	ELISA	UL HASSAN, Z., AL THANI, R., BALMAS, V., MIGHELI, Q. & JAOUA, S. 2019. Prevalence of Fusarium fungi and their toxins in marketed feed. Food Control, 104, 224-230.
Hassan	0	2019	Millet	Zearalenone			51		143	7	3	43	Asia	Qatar	1.75	0	ELISA	UL HASSAN, Z., AL THANI, R., BALMAS, V., MIGHELI, Q. & JAOUA, S. 2019. Prevalence of

																		Fusarium fungi and their toxins in marketed feed. Food Control, 104, 224-230.
Hassan	0	2019	Millet	Deoxynivalenol			37		37	7	1	14	Asia	Qatar	18.5	0	ELISA	UL HASSAN, Z., AL THANI, R., BALMAS, V., MIGHELI, Q. & JAOUA, S. 2019. Prevalence of Fusarium fungi and their toxins in marketed feed. Food Control, 104, 224-230.
Hassan	0	2019	Millet	Fumonisins			38		45	7	2	29	Asia	Qatar	25	0	ELISA	UL HASSAN, Z., AL THANI, R., BALMAS, V., MIGHELI, Q. & JAOUA, S. 2019. Prevalence of Fusarium fungi and their toxins in marketed feed. Food Control, 104, 224-230.
Hassan	0	2019	Barley	Zearalenone			0		0	4	0	0	Asia	Qatar	1.75	0	ELISA	UL HASSAN, Z., AL THANI, R., BALMAS, V., MIGHELI, Q. & JAOUA, S. 2019. Prevalence of Fusarium fungi and their toxins in marketed feed. Food Control, 104, 224-230.
Hassan	0	2019	Barley	Deoxynivalenol			48		75	4	2	50	Asia	Qatar	18.5	0	ELISA	UL HASSAN, Z., AL THANI, R., BALMAS, V., MIGHELI, Q. & JAOUA, S. 2019. Prevalence of Fusarium fungi and their toxins in marketed feed. Food Control, 104, 224-230.
Hassan	0	2019	Barley	Fumonisins			553		553	4	1	25	Asia	Qatar	25	0	ELISA	UL HASSAN, Z., AL THANI, R., BALMAS, V., MIGHELI, Q. & JAOUA, S. 2019. Prevalence of Fusarium fungi and their toxins in marketed feed. Food Control, 104, 224-230.
Hassan	0	2019	Soybeans	Zearalenone			0		0	6	0	0	Asia	Qatar	1.75	0	ELISA	UL HASSAN, Z., AL THANI, R., BALMAS, V., MIGHELI, Q. & JAOUA, S. 2019. Prevalence of Fusarium fungi and their toxins in marketed feed. Food Control, 104, 224-230.
Hassan	0	2019	Soybeans	Deoxynivalenol			38		49	6	4	67	Asia	Qatar	18.5	0	ELISA	UL HASSAN, Z., AL THANI, R., BALMAS, V., MIGHELI, Q. & JAOUA, S. 2019. Prevalence of Fusarium fungi and their toxins in marketed feed. Food Control, 104, 224-230.
Hassan	0	2019	Soybeans	Fumonisins			0		0	6	0	0	Asia	Qatar	25	0	ELISA	UL HASSAN, Z., AL THANI, R., BALMAS, V., MIGHELI, Q. & JAOUA, S. 2019. Prevalence of Fusarium fungi and their toxins in marketed feed. Food Control, 104, 224-230.
Murshed	0	2019	Soybeans	Total Aflatoxins	7.000	0.868	4.13	1	30.2	65	47	72	Asia	Yemen	0	0	ELISA	MURSHED, S. A. A., BACHA, N. & ALHARAZI, T. 2019. Detection of Total Aflatoxins in Groundnut and Soybean Samples in Yemen Using Enzyme-Linked Immunosorbent Assay. Journal of Food Quality.
Juan	2019	2019	Feed	Aflatoxin B1			0		0	122	0	0	Africa	Tunisia	1.68	5.6	LC-MC	JUAN, C., OUESLATI, S., MANES, J. & BERRADA, H. 2019. Multimycotoxin Determination in Tunisian Farm Animal Feed. Journal of Food Science, 84, 3885-3893.
Juan	2019	2019	Feed	Aflatoxin B2			0		0	122	0	0	Africa	Tunisia	1.84	6.13	LC-MC	JUAN, C., OUESLATI, S., MANES, J. & BERRADA, H. 2019. Multimycotoxin Determination in Tunisian Farm Animal Feed. Journal of Food Science, 84, 3885-3893.
Juan	2019	2019	Feed	Aflatoxin G1			0		0	122	0	0	Africa	Tunisia	1.59	5.29	LC-MC	JUAN, C., OUESLATI, S., MANES, J. & BERRADA, H. 2019. Multimycotoxin Determination in Tunisian Farm Animal Feed. Journal of Food Science, 84, 3885-3893.

Juan	2019	2019	Feed	Aflatoxin G2			8.49		42.47	122	1	1	Africa	Tunisia	2.09	7.64	LC-MC	JUAN, C., OUESLATI, S., MANES, J. & BERRADA, H. 2019. Multimycotoxin Determination in Tunisian Farm Animal Feed. Journal of Food Science, 84, 3885-3893.
Juan	2019	2019	Feed	Ochratoxin A	0.520	0.047	1.92		6.69	122	7	6	Africa	Tunisia	0.84	2.81	LC-MC	JUAN, C., OUESLATI, S., MANES, J. & BERRADA, H. 2019. Multimycotoxin Determination in Tunisian Farm Animal Feed. Journal of Food Science, 84, 3885-3893.
Juan	2019	2019	Feed	Deoxynivalenol	16.500	1.494	94.81		249	122	75	61	Africa	Tunisia	1.31	4.38	GC-MC	JUAN, C., OUESLATI, S., MANES, J. & BERRADA, H. 2019. Multimycotoxin Determination in Tunisian Farm Animal Feed. Journal of Food Science, 84, 3885-3893.
Juan	2019	2019	Feed	T-2 toxin	7.380	0.668	202.28		956	122	5	4	Africa	Tunisia	4.36	8.53	GC-MC	JUAN, C., OUESLATI, S., MANES, J. & BERRADA, H. 2019. Multimycotoxin Determination in Tunisian Farm Animal Feed. Journal of Food Science, 84, 3885-3893.
Juan	2019	2019	Feed	HT-2 toxin	10.020	0.907	34.4		119.2	122	24	20	Africa	Tunisia	1.97	6.55	GC-MC	JUAN, C., OUESLATI, S., MANES, J. & BERRADA, H. 2019. Multimycotoxin Determination in Tunisian Farm Animal Feed. Journal of Food Science, 84, 3885-3893.
Juan	2019	2019	Feed	Zearalenone	6.230	0.564	19.13		77.4	122	4	3	Africa	Tunisia	12.94	3.43	LC-MC	JUAN, C., OUESLATI, S., MANES, J. & BERRADA, H. 2019. Multimycotoxin Determination in Tunisian Farm Animal Feed. Journal of Food Science, 84, 3885-3893.
Iram	2015-2016	2019	Poultry Feed	Total Aflatoxins	24.281	1.700	25.51	4.23	72.27	204	132	65	Asia	Pskistan	0	0	ELISA	IRAM, S., FAREED, S. K., CHAUDHARY, M., IQBAL, M. U. N., GHANI, R., KHAN, T. A. & ABBAS, T. 2019. Identification of Aspergillus flavus and aflatoxin in home mix layer poultry feed in relation to seasons in Karachi, Pakistan. Tropical Animal Health and Production, 51, 1321-1327.
Aballah	2014-2015	2019	Maize	Aflatoxin B1			8.7	0.2	44.9	61	15	25	Africa	Egypt	0.04-0.12	0.12-0.39	HPLC	ABDALLAH, M. F., GIRGIN, G. & BAYDAR, T. 2019. Mycotoxin Detection in Maize, Commercial Feed, and Raw Dairy Milk Samples from Assiut City, Egypt. Vet Sci, 6.
Aballah	2014-2016	2019	Maize	Aflatoxin B2			2.2	0.1	7	61	6	10	Africa	Egypt	0.04-0.13	0.12-0.40	HPLC	ABDALLAH, M. F., GIRGIN, G. & BAYDAR, T. 2019. Mycotoxin Detection in Maize, Commercial Feed, and Raw Dairy Milk Samples from Assiut City, Egypt. Vet Sci, 6.
Aballah	2014-2017	2019	Maize	Aflatoxin G1			0		0	61	0	0	Africa	Egypt	0	0	HPLC	ABDALLAH, M. F., GIRGIN, G. & BAYDAR, T. 2019. Mycotoxin Detection in Maize, Commercial Feed, and Raw Dairy Milk Samples from Assiut City, Egypt. Vet Sci, 6.
Aballah	2014-2018	2019	Maize	Aflatoxin G2			0		0	61	0	0	Africa	Egypt	0	0	HPLC	ABDALLAH, M. F., GIRGIN, G. & BAYDAR, T. 2019. Mycotoxin Detection in Maize, Commercial Feed, and Raw Dairy Milk Samples from Assiut City, Egypt. Vet Sci, 6.
Aballah	2014-2019	2019	Maize	Ochratoxin A			0		0	61	0	0	Africa	Egypt	0	0	HPLC	ABDALLAH, M. F., GIRGIN, G. & BAYDAR, T. 2019. Mycotoxin Detection in Maize, Commercial Feed, and Raw Dairy Milk Samples from Assiut City, Egypt. Vet Sci, 6.

Aballah	2014-2020	2019	Maize	Zearalenone			0		0	61	0	0	Africa	Egypt	0	0	HPLC	ABDALLAH, M. F., GIRGIN, G. & BAYDAR, T. 2019. Mycotoxin Detection in Maize, Commercial Feed, and Raw Dairy Milk Samples from Assiut City, Egypt. Vet Sci, 6.
Aballah	2014-2015	2019	Feed	Aflatoxin B1			1.5	0.1	5.9	17	8	47	Africa	Egypt	0.04-0.12	0.12-0.39	HPLC	ABDALLAH, M. F., GIRGIN, G. & BAYDAR, T. 2019. Mycotoxin Detection in Maize, Commercial Feed, and Raw Dairy Milk Samples from Assiut City, Egypt. Vet Sci, 6.
Aballah	2014-2016	2019	Feed	Aflatoxin B2			0.5		0.5	17	1	6	Africa	Egypt	0.04-0.13	0.12-0.40	HPLC	ABDALLAH, M. F., GIRGIN, G. & BAYDAR, T. 2019. Mycotoxin Detection in Maize, Commercial Feed, and Raw Dairy Milk Samples from Assiut City, Egypt. Vet Sci, 6.
Aballah	2014-2017	2019	Feed	Aflatoxin G1			0		0	17	0	0	Africa	Egypt	0	0	HPLC	ABDALLAH, M. F., GIRGIN, G. & BAYDAR, T. 2019. Mycotoxin Detection in Maize, Commercial Feed, and Raw Dairy Milk Samples from Assiut City, Egypt. Vet Sci, 6.
Aballah	2014-2018	2019	Feed	Aflatoxin G2			0		0	17	0	0	Africa	Egypt	0	0	HPLC	ABDALLAH, M. F., GIRGIN, G. & BAYDAR, T. 2019. Mycotoxin Detection in Maize, Commercial Feed, and Raw Dairy Milk Samples from Assiut City, Egypt. Vet Sci, 6.
Aballah	2014-2019	2019	Feed	Ochratoxin A			0		0	17	0	0	Africa	Egypt	0	0	HPLC	ABDALLAH, M. F., GIRGIN, G. & BAYDAR, T. 2019. Mycotoxin Detection in Maize, Commercial Feed, and Raw Dairy Milk Samples from Assiut City, Egypt. Vet Sci, 6.
Aballah	2014-2020	2019	Feed	Zearalenone			8.1	1	11.9	17	4	24	Africa	Egypt	0.92	2.8	HPLC	ABDALLAH, M. F., GIRGIN, G. & BAYDAR, T. 2019. Mycotoxin Detection in Maize, Commercial Feed, and Raw Dairy Milk Samples from Assiut City, Egypt. Vet Sci, 6.
shar	2019	2020	Cotton seed	Aflatoxin B1	2.770	0.264	41		90	110	88	80	Asia	Pakistan	0.25	0.71	ELISA	SHAR, Z. H., PIRKASH, O., SHAR, H. H., SHERAZI, S. T. H. & MAHESAR, S. A. 2020. Aflatoxins in cotton seeds and cotton seed cake from Pakistan. Food Addit Contam Part B Surveill, 13, 72-76.
shar	2019	2020	Cotton seed	Aflatoxin G1	3.160	0.301	7		10	110	56	51	Asia	Pakistan	0.16	0.47	ELISA	SHAR, Z. H., PIRKASH, O., SHAR, H. H., SHERAZI, S. T. H. & MAHESAR, S. A. 2020. Aflatoxins in cotton seeds and cotton seed cake from Pakistan. Food Addit Contam Part B Surveill, 13, 72-76.
shar	2019	2020	Cotton seed	Aflatoxin B2	3.280	0.313	16		31	110	66	60	Asia	Pakistan	0.3	0.9	ELISA	SHAR, Z. H., PIRKASH, O., SHAR, H. H., SHERAZI, S. T. H. & MAHESAR, S. A. 2020. Aflatoxins in cotton seeds and cotton seed cake from Pakistan. Food Addit Contam Part B Surveill, 13, 72-76.
shar	2019	2020	Cotton seed	Aflatoxin G2	3.220	0.307	5		8	110	33	30	Asia	Pakistan	0.2	0.58	ELISA	SHAR, Z. H., PIRKASH, O., SHAR, H. H., SHERAZI, S. T. H. & MAHESAR, S. A. 2020. Aflatoxins in cotton seeds and cotton seed cake from Pakistan. Food Addit Contam Part B Surveill, 13, 72-76.
shar	2019	2020	Cotton seed cake	Aflatoxin B1	2.990	0.285	43		150	110	97	88	Asia	Pakistan	0.3	0.91	ELISA	SHAR, Z. H., PIRKASH, O., SHAR, H. H., SHERAZI, S. T. H. & MAHESAR, S. A. 2020. Aflatoxins in cotton seeds and cotton seed cake

																		from Pakistan. Food Addit Contam Part B Surveill, 13, 72-76.
shar	2019	2020	Cotton seed cake	Aflatoxin G1	3.360	0.320	10		20	110	58	53	Asia	Pakistan	0.28	0.88	ELISA	SHAR, Z. H., PIRKASH, O., SHAR, H. H., SHERAZI, S. T. H. & MAHESAR, S. A. 2020. Aflatoxins in cotton seeds and cotton seed cake from Pakistan. Food Addit Contam Part B Surveill, 13, 72-76.
shar	2019	2020	Cotton seed cake	Aflatoxin B2	3.400	0.324	29		81	110	84	76	Asia	Pakistan	0.19	0.57	ELISA	SHAR, Z. H., PIRKASH, O., SHAR, H. H., SHERAZI, S. T. H. & MAHESAR, S. A. 2020. Aflatoxins in cotton seeds and cotton seed cake from Pakistan. Food Addit Contam Part B Surveill, 13, 72-76.
shar	2019	2020	Cotton seed cake	Aflatoxin G2	3.210	0.306	7		18	110	62	56	Asia	Pakistan	0.1	0.3	ELISA	SHAR, Z. H., PIRKASH, O., SHAR, H. H., SHERAZI, S. T. H. & MAHESAR, S. A. 2020. Aflatoxins in cotton seeds and cotton seed cake from Pakistan. Food Addit Contam Part B Surveill, 13, 72-76.
juan	2018	2020	silage	HT-2 toxin	78.000	13.789	5.3		116	32	3	9	Africa	Tunisia	1.97	6.55	GC-MC	JUAN, C., MANNAI, A., BEN SALEM, H., OUESLATI, S., BERRADA, H., JUAN-GARCIA, A. & MANES, J. 2020. Mycotoxins presence in pre- and post-fermented silage from Tunisia. Arabian Journal of Chemistry, 13, 6753-6761.
juan	2018	2020	silage	Deoxynivalenol	538.000	95.106	153.9		915.9	32	9	28	Africa	Tunisia	1.31	4.38	GC-MC	JUAN, C., MANNAI, A., BEN SALEM, H., OUESLATI, S., BERRADA, H., JUAN-GARCIA, A. & MANES, J. 2020. Mycotoxins presence in pre- and post-fermented silage from Tunisia. Arabian Journal of Chemistry, 13, 6753-6761.
Waqas	2017-2018	2021	Mustard oil cake	Aflatoxin B1	4.500	0.795	25	0.03	150.8	32	22	69	Asia	Pakistan	0.03	0.1	HPLC-FLD	WAQAS, M., PERVAIZ, W., ZIA, K. M. & IQBAL, S. Z. 2021. Assessment of aflatoxin B-1 in animal feed and aflatoxin M-1 in raw milk samples of different species of milking animals from Punjab, Pakistan. Journal of Food Safety, 41.
Waqas	2017-2018	2021	Soybean oil cake	Aflatoxin B2	4.500	0.822	13.5	0.03	120.5	30	20	67	Asia	Pakistan	0.03	0.1	HPLC-FLD	WAQAS, M., PERVAIZ, W., ZIA, K. M. & IQBAL, S. Z. 2021. Assessment of aflatoxin B-1 in animal feed and aflatoxin M-1 in raw milk samples of different species of milking animals from Punjab, Pakistan. Journal of Food Safety, 41.
Waqas	2017-2018	2021	Corn oil cake	Aflatoxin B3	6.700	1.117	20.1	0.03	180.5	36	19	53	Asia	Pakistan	0.03	0.1	HPLC-FLD	WAQAS, M., PERVAIZ, W., ZIA, K. M. & IQBAL, S. Z. 2021. Assessment of aflatoxin B-1 in animal feed and aflatoxin M-1 in raw milk samples of different species of milking animals from Punjab, Pakistan. Journal of Food Safety, 41.
Waqas	2017-2018	2021	Sunflower oil cake	Aflatoxin B4	4.900	0.828	17.2	0.03	110.5	35	25	71	Asia	Pakistan	0.03	0.1	HPLC-FLD	WAQAS, M., PERVAIZ, W., ZIA, K. M. & IQBAL, S. Z. 2021. Assessment of aflatoxin B-1 in animal feed and aflatoxin M-1 in raw milk samples of different species of milking animals from Punjab, Pakistan. Journal of Food Safety, 41.
Waqas	2017-2018	2021	Cotton seeds	Aflatoxin B5	2.800	0.560	17.7	0.03	180.5	25	19	76	Asia	Pakistan	0.03	0.1	HPLC-FLD	WAQAS, M., PERVAIZ, W., ZIA, K. M. & IQBAL, S. Z. 2021. Assessment of aflatoxin B-1 in animal feed and aflatoxin M-1 in raw milk samples of

																		different species of milking animals from Punjab, Pakistan. Journal of Food Safety, 41.
Waqas	2017-2018	2021	Dry wheat bread	Aflatoxin B6	5.700	0.963	15.2	0.03	80.5	35	21	60	Asia	Pakistan	0.03	0.1	HPLC-FLD	WAQAS, M., PERVAIZ, W., ZIA, K. M. & IQBAL, S. Z. 2021. Assessment of aflatoxin B-1 in animal feed and aflatoxin M-1 in raw milk samples of different species of milking animals from Punjab, Pakistan. Journal of Food Safety, 41.
